# Conservation of the dehiscence zone gene regulatory network in dicots and the role of the *SEEDSTICK* ortholog of California poppy (*Eschscholzia californica*) in fruit development

**DOI:** 10.1186/s13227-024-00236-0

**Published:** 2024-12-27

**Authors:** Dominik Lotz, Le Han Rössner, Katrin Ehlers, Doudou Kong, Clemens Rössner, Oliver Rupp, Annette Becker

**Affiliations:** 1 Institute of Botany, Justus-Liebig-University, Heinrich-Buff-Ring 38, 35392 Giessen, Germany; 2 Bioinformatics and Systems Biology, Justus-Liebig-University, Heinrich-Buff-Ring 58, 35392 Giessen, Germany

## Abstract

**Background:**

Fruits, with their diverse shapes, colors, and flavors, represent a fascinating aspect of plant evolution and have played a significant role in human history and nutrition. Understanding the origins and evolutionary pathways of fruits offers valuable insights into plant diversity, ecological relationships, and the development of agricultural systems. *Arabidopsis thaliana* (Brassicaceae, core eudicot) and *Eschscholzia californica* (California poppy, Papaveraceae, sister group to core eudicots) both develop dry dehiscent fruits, with two valves separating explosively from the replum-like region upon maturation. This led to the hypothesis, that homologous gene regulatory networks direct fruit development and dehiscence in both species.

**Results:**

Transcriptome analysis of separately collected valve and replum-like tissue of California poppy yielded the S*EEDSTICK* (*STK)* ortholog as candidate for dehiscence zone regulation. Expression analysis of *STK* orthologs from dry dehiscing fruits of legumes (*Vicia faba*, *Glycine max* and *Pisum sativum)* shows their involvement in fruit development. Functional analysis using Virus-Induced Gene Silencing (VIGS) showed premature rupture of fruits and clarified the roles of *EscaSTK*: an evolutionary conserved role in seed filling and seed coat development, and a novel role in restricting cell divisions in the inner cell layer of the valve.

**Conclusion:**

Our analysis shows that the gene regulatory network described in Arabidopsis is significantly different in other dicots, even if their fruits form a dehiscence zone at the valve margins. The ortholog of *STK*, known to be involved in ovule development and seed abscission in Arabidopsis, was recruited to a network regulating fruit wall proliferation in California poppy. There, *EscaSTK* allows fruit maturation without premature capsule rupture, highlighting the importance of proper endocarp development for successful seed dispersal.

**Supplementary Information:**

The online version contains supplementary material available at 10.1186/s13227-024-00236-0.

## Background

All flowering plants develop fruits that house the seeds and ensure dispersal of the mature seeds. The evolutionary innovation of carpels that, after fertilization mature into fruits, provided angiosperms with a competitive advantage, allowing them to colonize diverse habitats and establish complex ecological relationships with animals. Some fruits make use of wind, water or animals to move their fruits and seeds away from the maternal plant. Flowering plants utilize a large diversity of mechanisms to disperse their seeds, often involving intricate morphological adaptations, such as feathery appendages, floating devices or hooks. Seed dispersal mechanisms are important traits when considering biodiversity conservation because it enables colonization of new habitats ensuring species survival [2, 30]. Understanding dehiscence as seed-dispersal mechanism is also of economic interest. Many crops, such as *Brassica napus* (canola) or the protein-rich legumes like soy (*Glycine max*), pea (*Pisum sativum*) or lentil (*Lens culinaris*) have dry dehiscent fruits and pre-harvest fruit dehiscence causes major yield loss [43]. Therefore, identifying key regulators involved in dehiscence zone development has been a major focus in the past.

Dry dehiscent fruits shatter upon maturity to disperse the seeds, often explosively, requiring the coordination of fruit opening and seed detachment from the fruit [2]. In *Arabidopsis* fruits, the two valves separate from the replum at the dehiscence zone (DZ) that runs along the full length of the fruit [13, 15]. The DZ is formed by a precise patterning of lignified and unlignified cells at the replum/valve margins. The fruits break open when the combination of those two cell types builds up tension resulting from the differential hygro-responsive properties upon drying out [3]. This process also involves weakening of cell adhesion at the separation layer with tensions provided by the surrounding tissues or external agents. The mentioned tensions can come from pod walls, properties of lignified and non-lignified tissues or changes in turgor association [3, 22].

**Fig. 1:**
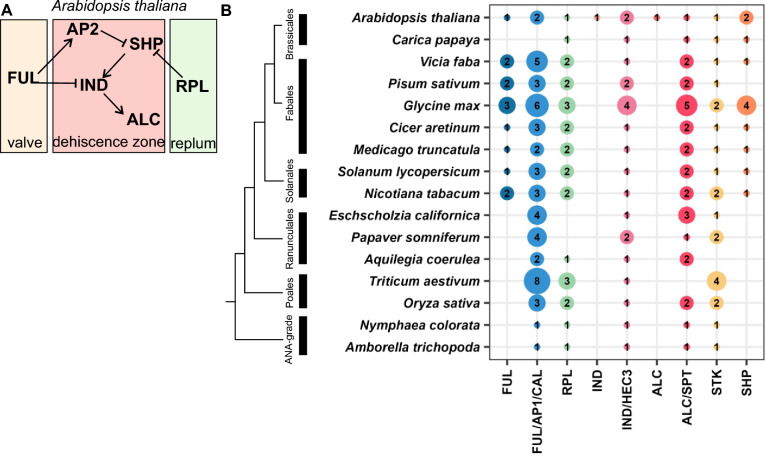
Regulatory network of DZ formation in *A. thaliana* and presence of orthologs of DZ regulatory transcription factors in other plant species. **A** Simplified representation of the known transcription factors important for DZ formation in *Arabidopsis thaliana* based on Ballester and Ferrandiz, 2017. Gene activation is indicated by arrows, repression by bars. **B** Summary of the phylogeny reconstructions of a subset of transcription factor families shown in A (Suppl. Figure 1). On the left, a simplified phylogeny shows the relationship of the species included in our analysis. Numbers in colored bubbles represent copy numbers of the respective homologs. In addition to the single *FUL* and *ALC* column, a *FUL/AP/CAL* and *ALC/SPT* clades are generated due to an unclear phylogenetic separation between these genes (Suppl. Figure 1)

The gene regulatory network (GRN) directing fruit tissue specification and DZ development in the dry dehiscent species *Arabidopsis* was elucidated previously (Fig. 1A): the formation of the DZ with its intricate lignin pattern is regulated by *SHATTERPROOF1* (*SHP1*) and *SHATTERPROOF2* (*SHP2*), *INDEHISCENT* (*IND*) and *ALCATRAZ* (*ALC*). *ALC* is required for the formation of the separation layer, composed of small cells with only little cell wall lignification at the valve/replum border. In Solanaceae species, *ALC* orthologs are involved in repressing lignification during fruit development [38]. *SHP1* and *SHP2* induce *IND,* which activates lignin biosynthesis genes in the DZ [3, 28, 29, 49].

*FRUITFULL* (*FUL*) specifies valve identity and *REPLUMLESS* (*RPL*) specifies replum identity (Fig. 1A), limiting the DZ region to the valve/replum margins [14, 29, 51]. Interestingly, indehiscence can also be achieved in the dry dehiscent species *Nicotiana benthamiana* when the *FUL* or *SHP* orthologs are knocked down, suggesting some degree of functional conservation of these two genes among dicots [3].

Little is known about the extent of conservation of fruit DZ regulatory genes in dicots. Dry dehiscent fruits have in common that their fruit walls are comparatively thin, as the units of propagation are the released seeds. Thus, a tight regulation of fruit wall thickness is required as fruits with excessive internal tissue may rupture prematurely.

Interestingly, the GRN regulating lengthwise fruit expansion requires *STK* in Arabidopsis, which activates cytokinine-degrading enzymes and *FUL*, both of which are required for fruit elongation. This deprives the developing fruit of cytokinine, which is required before fertilization for gynoecium formation [10]. After fertilization, lengthwise cell divisions are infrequent and most of the valve elongation occurs via cell expansion and is limited to the regions harboring fertilized ovules, suggesting a mobile, seed-derived signaling cascade [50]. However, little is known about the genetic regulation of fruit expansion along the horizontal plane in *Arabidopsis*, aside from those genes that are also involved in adaxial/abaxial polarity specification in lateral organs, such as *KANADI*, *GYMNOS*, or *YABBY* family genes. However, their mutant’s phenotypes are not affected in the number of cell layers in cross sections [12]. Comparing dry and dehiscent fruits in the Solanaceae, Pabon-Mora and Litt (2011) documented that postfertilization fruit wall expansion can be driven by cell divisions of all layers of the fruit wall, exo-, endo-, and mesocarp, and phylogenetic aspects seem to play a more important role than fruit types in determining which layer expands most.

The purpose of this study was to find candidate regulators of dry dehiscent fruit development in species outside the core eudicots. The Papaveraceae family, in particular, exhibits remarkable diversity in dry dehiscent fruit types, such as the poricidal capsule of Opium poppy (*Papaver somniferum*) or the longitudinally dehiscent fruits of California poppy (*Eschscholzia californica*) [25]. This may help to elucidate how fruits of species develop whose genomes do not encode orthologs of the well-known *Arabidopsis* fruit development genes. Therefore, California poppy has been proven to be a suitable model plant for studying the GRN of DZ patterning, owing to its homology to the fruit of *Arabidopsis*. California poppy has been used as a genetic model system to compare gene functions within the dicots, mainly because of its phylogenetic position as a representative of the sister lineage to the core eudicots and its amenability to Virus-Induced-Gene Silencing (VIGS) [4, 6]. Similar to *Arabidopsis*, the fruit is composed of two valves connected to a persistent medial tissue, similar to the replum, with the valves explosively separating from the replum-like tissue upon dehydration during maturity [5, 41].

This study addresses the following questions: (1) to which extent is the dehiscence zone GRN network conserved in angiosperms? (2) Which transcription factors may contribute to DZ formation in California poppy? (3) What is the role of *STK* in California poppy fruit development? (4) Are *STK* homologs differentially expressed in valve and DZ of fruits in other eudicots?

## Results

### Most homologs of *Arabidopsis* fruit dehiscence regulators are absent from the *E. californica* genome

The *Arabidopsis* DZ GRN is well documented (Fig. 1A), but we were interested to learn about its degree of conservation across angiosperms. In the likely case that the major regulators of DZ formation are absent in species outside the Brassicaceae, DZs in other species are patterned by different networks. These may only partly rely on orthologs known from *Arabidopsis*. We carried out phylogeny reconstructions including representatives of major angiosperm lineages with sequenced genomes for homologs of transcription factors constituting the core GRN of *A. thaliana* (Fig. 1A, Supplemental Fig. 1). These revealed only partial conservation, with DZ regulator homologs missing from many lineages (Fig. 1B). *RPL* is known to be involved in replum specification and homologs of *RPL* are found in all angiosperms, most species maintaining more than one copy. However, extensive phylogenetic analyses show that California poppy does not encode for *RPL *[63] Furthermore, *IND* is found only in *Arabidopsis*, suggesting specificity to Brassicaceae, while *ALC* is present in core eudicots. *ALC* and *SPT* result from a gene duplication of an *ALC/SPT*-like gene in the lineage leading to core eudicots, with the *ALC* coding sequence lacking several conserved segments, as also shown previously by others [17, 62]. *ALC/SPT*-like genes can be found in almost all species analyzed, most retaining more than one copy. *FUL1* orthologs are found in most species analyzed but are lacking in the Ranunculales, indicating these orthologs derived from a core eudicot specific duplication. *SHP* genes are present in the core eudicots only, but even in this lineage, they are absent from *Pisum sativum* (pea). In contrast, *STK* homologs are found in all species analyzed, with the exception of *Aquilegia coerulea* (columbine) (Fig. 1B, [41]). Interestingly, *Amborella trichopoda* and *Nymphaea colorata*, from the ANA grade, have only a single homolog of *Arabidopsis* DZ regulator genes. In contrast, species with notoriously large genomes like wheat and soybean have retained many copies of these genes, for example eight *FUL*-like genes in wheat. In California poppy, the DZ GRN lacks the orthologs of DZ and replum regulators, such as *RPL*, *IND*, and *SHP1/2*, while *FUL/AP1/CAL, IND/HEC3, ALC/SPT,* and *STK* homologs are present. Taken together, the specification of the California poppy DZ requires a set of regulators at least partially different from the ones characterized in *Arabidopsis*.

### Fruit anatomy and differential gene expression of valve and replum-like tissue in California poppy fruits

**Fig. 2:**
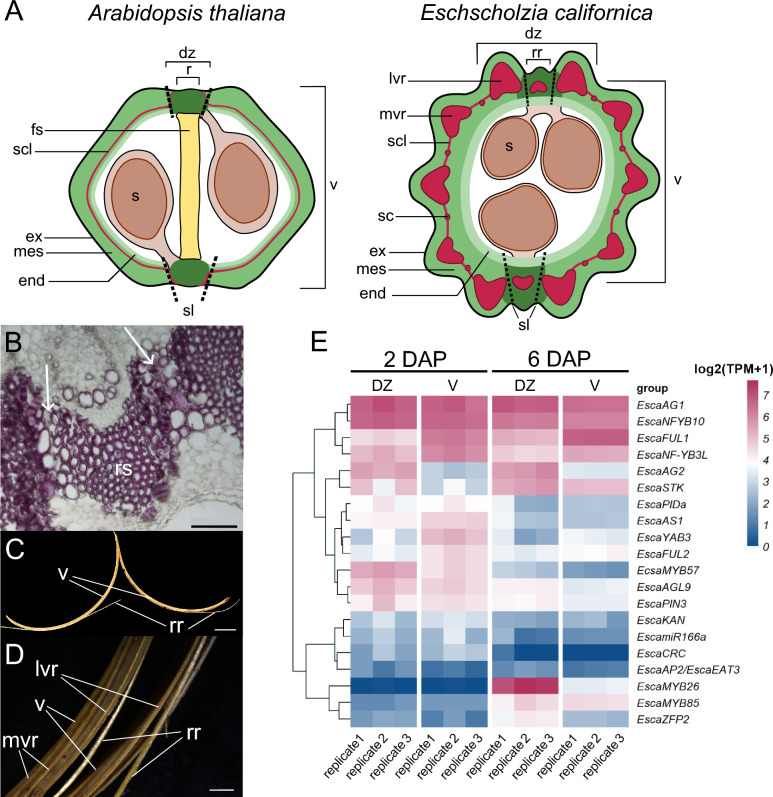
Comparative schematic drawing of *Arabidopsis* and California poppy fruit, fruit opening and transcriptome analysis of DZ and valves of California poppy. **A** Schematic overview of Arabidopsis (left) and California poppy (right) fruit cross-section, red represents lignified tissues, while green color indicates the pericarp that comprises exocarp (ex), mesocarp (mes), endocarp (end). Both fruits are composed of two valves (v), separated by a replum (r) or replum-like region (rr) including, dehiscence zone (dz), dashed lines show separation layer (sl). Seeds are attached at the placenta, which is connected to the r or rr, respectively. In Arabidopsis, the lignified sclerified layer (scl) stretches along the endocarp, while in California poppy, the scl is interspersed by lateral valve ridges (lvr) median valve ridges (mvr) with sclerified cells (sc) in between. *Arabidopsis* fruit is separated by a false septum (fs), a structure lacking in California poppy. **B** Enlargement of the dehiscence zone showing the replum sclerenchyma and lignified cell files that will separate upon rupture (white arrows), scale bar represents 100 µm. **C** Split fruit after rupture with valves (V) and fragments of replum-like region (rr) indicated. **D** Enlargement of ruptured valves. **E** Transcriptome analysis of 2 dap (young) and 6 dap (late) stage of fruit development showing dehiscence zones separated from valve regions. Clustering was done using Euclidean distance. Expression is shown as log2(TPM + 1) Scale bar represents 100 µm in B, 1 cm in C and 2 mm in D

The California poppy fruit anatomy is reminiscent of that from *Arabidopsis*: two congenitally fused carpels form the gynoecium, and the fruit is dry dehiscent, with a clear differentiation of valve, replum-like region and DZ (Fig. 2A). The replum-like region is a persistent medial tissue that remains after seed dispersal as a thin, visible layer between the valves. The funiculus of the seed-bearing ovule is attached to the placenta located at the replum-like region. Within the locule, two to three round-shaped seeds are present (Supplemental Fig. 2Q). However, the fruit is more rigid than that of *Arabidopsis* due to thick lignified lateral ridges along the valve. The endocarp layer is composed of large cells with thick tangential walls and remains unlignified. Endodermal cells along with adjacent parenchyma cells collapse during fruit maturation and desiccation (for a detailed description of fruit and DZ development, including a timeline for lignification, see Suppl. Material 1 and Suppl. Figure 2). Interestingly, its DZ architecture deviates strongly from that of *Arabidopsis*: shortly before dehiscence, around 32 days after pollination (dap), each valve shows five strongly lignified ridges abaxially of the vascular bundles. A sclerenchymatic ring of lignified cells loosely connects these ridges, forming a rather zigzag-shaped circle. The two lateral valve ridges are more elevated and part of the fully lignified DZ, which resides at the connection of the two valves (Fig. 2A). The replum-like region is composed of a half-moon shaped sclerenchyma cap on top of a vascular bundle (Fig. 2B). The fruits will eventually dehisce explosively in acropetal direction starting from the base up to the style. The valves detach at the lignified separation layer between the replum-like region and the two lateral valve ridges (Fig. 2C, D) but remain connected at the fruit apex after dehiscence.

To identify candidate genes for DZ regulation in California poppy, we hand-dissected fruits early in their development at two distinct developmental stages. DZ including replum-like region and lateral valve ridges were collected separately from the valve tissue, while avoiding contamination with placenta tissue and developing ovules. At 2 dap (young stage, Suppl. Figure 2 A, B), clusters of future sclerenchyma cells became visibly distinct from the surrounding parenchyma tissue at the future lateral and median valve ridges, and replum-like region, but lignification has not commenced yet. At 6 dap (later stage, Suppl. Figure 2 C, D), the sclerenchyma patterning is completed and lignification will commence shortly. The transcriptome analysis was carried out in three biological replicates with between 48 and 62 million reads per replicate. Figure 2E and Suppl. Figure 3 show expression of homologs of *Arabidopsis* DZ and other putative developmental regulators in the valve and replum-like region at two different stages. The two *FUL* paralogs, *EcFUL1* and *EcFUL2* show indeed stronger expression in the valves than in the DZ, with *EcFUL1* expressed at a higher level (Fig. 2E). The closest relatives of *Arabidopsis* DZ markers *SHP1* and *SHP2* in California poppy are *EscaAG1* and *EscaAG2*, and while *EscaAG1* is strongly expressed throughout all transcriptomes, *EscaAG2* shows stronger expression in the DZ than in the valve regions of both stages observed (Fig. 2E). However, the two *SPT* homologs of California poppy are not differentially expressed, as are the *AP2* homologs (Suppl. Figure 3).

In addition, we find that several genes whose Arabidopsis orthologs are not known to be involved in DZ formation show expression differences between the developmental stages (Fig. 2E), such as *EscaMYB85*, which is expressed stronger at 6 dap, and *EscaPIDa* and *EscaAS1*, which are expressed stronger at 2 dap. *EscaZFP2* and *EscaMYB26* show strong expression in only the 6 dap DZ. Only few genes like *EscaNF-YB3L, EscaAG2, EscaYAB3, EcFUL1* and *2* are expressed differentially between DZ and valve region in both stages and they may be involved in the continuous regulation of DZ patterning. *EscaSTK*, also known as *EscaAGL11*, is specifically down regulated in young valves at 2 dap suggesting a role in fruit development. Taken together, we find substantial differences in the expression of transcriptional regulators between the two developmental stages and valve and DZ tissues.

### Down regulation of *EscaSTK* affects floral organ identity and leads to premature fruit rupture

**Fig. 3:**
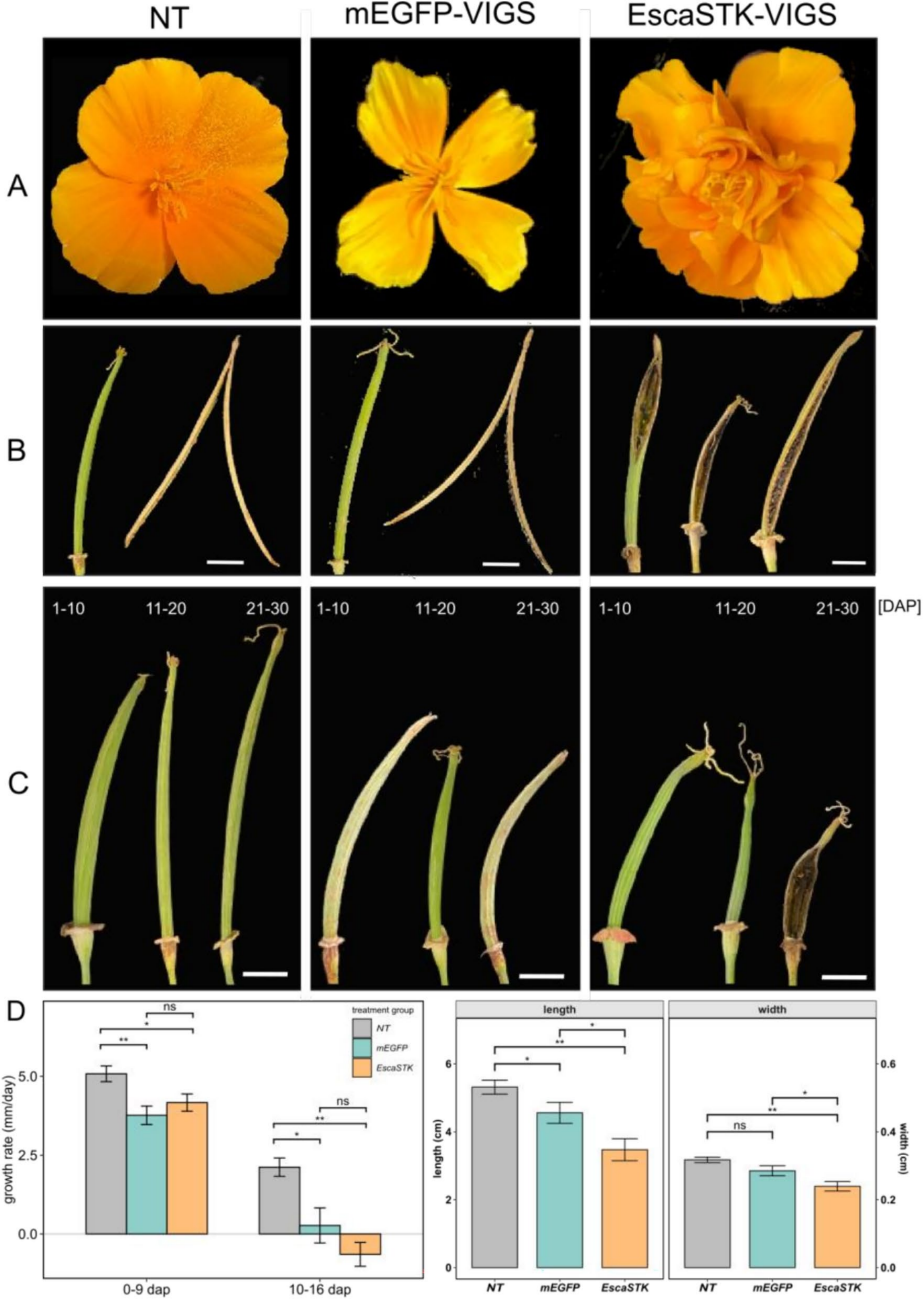
Effects of Virus-induced gene silencing of *EscaSTK* on flower and fruit development. **A** Effect of VIGS on flower morphology, with untreated plants (left), *mEGFP*-VIGS (center) and *EscaSTK*-VIGS (right) treated plants. **B** Fruits before desiccation and after dehiscence with *EscaSTK*-VIGS showing premature rupture before dehiscence. **C** Fruit morphology during different collection intervals (DAP). Scale bar represents 1 cm. **D** Left shows quantification of growth rates in mm per day of early (0–9 dap) and late (10–16 dap) timeframe, right shows maximal fruit size of length and width in cm. Calculation of significance was performed via Wilcoxon signed-rank test. ns: not significant, * p < 0.05, ** p < 0.01

*EscaSTK* was then functionally characterized as a candidate gene for a fruit developmental regulator, based on its specific expression pattern. VIGS was carried out to down regulate the expression of *EscaSTK*. 155 plants were treated with *EscaSTK*-VIGS and analyzed for phenotypic differences, together with with *EscaPDS-*VIGS treated plants for visual control of the experiment, *mEGFP*-VIGS as negative (mock) control, and untreated plants which were all compared with each other (Fig. 3, Table 1, Suppl. Figure 4). The first flower bud of the VIGS-treated plants was removed for RNA extraction and the three next formed flowers/fruits were analyzed. Almost all flowers of *EscaSTK*-VIGS resembled the untreated flower. However, in 6.5% of all *EscaSTK*-VIGS treated plants, homeotic conversions of outer stamen to petals were observed (Fig. 3A, Table 1). Interestingly, also the *mEGFP*-VIGS treated plants were affected in their flower development, with petals growing narrower when compared to the untreated plants (Fig. 3A). Vegetative development of *EscaSTK-* and *mEGFP*-VIGS treated plants was not affected. All *EscaPDS*-VIGS treated plants showed photobleaching in vegetative organs and flowers (Suppl. Figure 4).

**Table 1:** Numbers of VIGS-treated plants and phenotype occurrence

Treatment	No. of plants	No. of capsules	No. of plants not flowering	WT-like phenotype	Prematurely ruptured fruits	Flowers with homeotic conversions
Untreated	30	83	1 3%	30 100%	0 0%	0 0%
pTRV2-mEGFP	59	254	3 5,1%	56 94,9%	0 0%	0 0%
pTRV2-EscaSTK	155	521	13 8,4%	114 73,5%	18 11,6%	10 6,5%

VIGS treatment significantly reduces the growth rates of fruits in the rapid elongation phase between pollination and 9 dap, but also in the final, slower elongation phase between 10 and 16 dap (Fig. 3D). Fruits of *EscaSTK*- and *mEGFP*-VIGS treated failed to fully expand in length and width. Fruits of untreated plants reach around 6 cm in length and 3.5 mm in width. In contrast, *EscaSTK*- and *mEGFP*-VIGS treated plants grow only up to approximately 4.5 cm and 4.8 cm in width, respectively (Fig. 3D). While there is only little difference in fruit growth rate up to 9 dap, fruit growth of VIGS- treated plants differs significantly between 10 and 16 dap (Fig. 3D). Fruits of *mEGFP*-VIGS treated plants grow less than those of untreated plants and fruit growth rate of *EscaSTK*-VIGS treated plants becomes even negative after 10 dap. This may be due to a cessation of growth in combination with premature opening and dehydration.

Untreated (NT) and *mEGFP*-VIGS treated fruits open around 30 dap to explosively release the seeds (Fig. 3B). In 11.8% of the *EscaSTK*-treated plants, premature opening was observed (Table 1) already after on average 13 dap, resulting in fruits and seeds that dry out prematurely and failure to disperse the seeds (Fig. 3B and C). Our results suggest that *EscaSTK* contributes to a lesser extent to floral organ identity of stamens, with its main role is to prevent premature fruit rupture allowing for seed maturation and explosive seed dispersal.

### *EscaSTK* down regulation causes phenotypic alterations of the endocarp and seed morphology

**Fig. 4:**
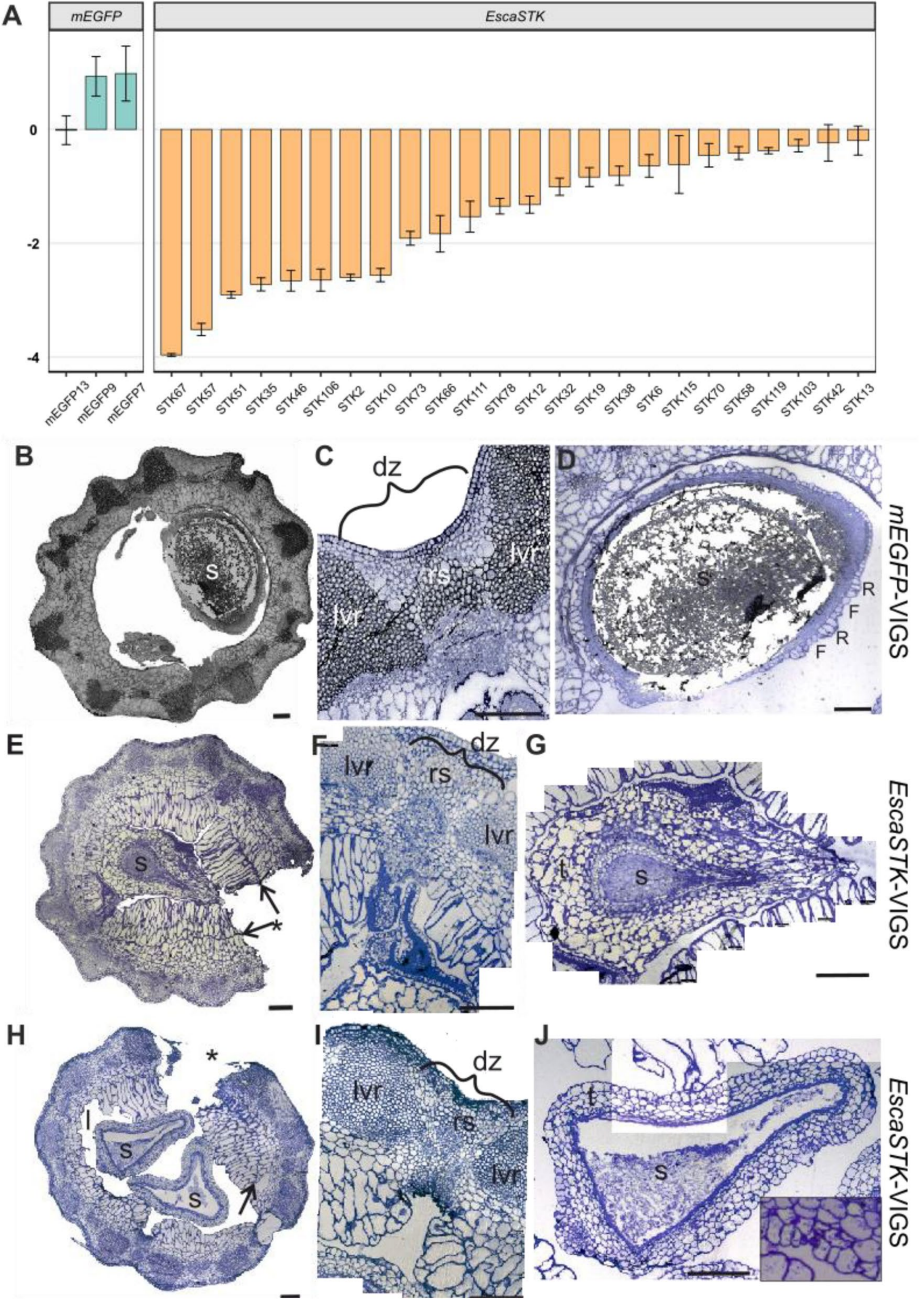
Down regulation of *EscaSTK* by VIGS and effects of *EscaSTK*-VIGS on tissue development of fruits and seeds. **A** qRT-PCR analysis of the first buds of VIGS-treated plants. Gene expression (Log_2_(2^∆∆Ct^)) relative to untreated plants is shown, with *GAPDH* as internal standard. **B, E, H** Overview of the capsule, **C, F, I** detailed view of the DZ, and **D, G, J** detailed view of the seed. **B-D** show a cross section of mEGFP-VIGS treated capsule as negative control with a WT-like histology. The respective images of prematurely opened *EscaSTK* capsules with intermediate down regulation (50–75% of remaining *EscaSTK* expression, **E**–**G**) or with severe down regulation (25–50% of remaining *EscaSTK* expression, **H**–**J**) are shown. Histology of the dehiscence zone and the abaxial valve tissues, as well as the early lignification events in the fruit wall, remain largely unaltered in *EscaSTK* capsules (**E**, **F**, **H**, **I**). Arrows in E and H denote the thick-walled tangential boundary between endocarp layers and underlying tissues. The asterisks in **H** and **E** indicated premature rupture of the capsule either at the dehiscence zone (**H**) or between the median valve ridges (**E**). The inset in J shows stomata in the seed epidermis. Scale bars are 200 µm. dz: dehiscence zone, F: facet cells, l: locule, lvr: lateral valve ridge, R: ridge cells, s: seed

*EscaSTK*-VIGS treated fruits show a high variance in length and width in addition to premature rupturing, and we were interested to learn if these phenotype observations depend on the degree of *EscaSTK* expression down regulation. qRT-PCR analysis with *EscaSTK*-specific primers not overlapping with the VIGS fragment was carried out in technical triplicates of three *mEGFP*- and 24 randomly selected *EscaSTK*-VIGS treated plants. One-third of the plants showed downregulation of *EscaSTK* to less than 50% of the wild type expression, suggesting that variance in expression may be related to variance in phenotype (Fig. 4A).

As the *EscaSTK*-VIGS treated plants show premature rupture of fruits (Fig. 3), we were interested to learn how the fruit tissues are affected by the reduction of *EscaSTK* expression. Cross sections of *mEGFP*-VIGS treated plants showed similar morphologies in overall architecture (Fig. 4B), dehiscence zone patterning (Fig. 4C), and seed morphology (Fig. 4D) to untreated plants (Fig. 2B). Figure 4E–G and Fig. 4H–J show two independently *EscaSTK*-VIGS treated plants with prematurely ruptured fruits. The most striking observation in those plants is the altered shape and size of the endocarp and adjacent fruit cell layers (Fig. 4E–I). In these tissues, the unlignified endocarp cells grew larger and elongated in radial direction. These elongated cell layers emerged at the valve margins and tapered towards the valve centers. However, they did not cover the replum-like region so that there was always a hollow slit between the elongated cells originating from the two valves (Fig. 4 E, H, F and I). The elongated cells filled almost the entire space between the growing seeds in the capsule, i.e., the former locule (Fig. 4H and E). Further, in *EscaSTK*-VIGS treated plants, the endocarp layer sometimes underwent additional, tangential cell divisions such that additional parenchymatous cell layers developed at the adaxial side of the pericarp (Fig. 4 E, H and Suppl. Figure 2 N-P, arrows). This additional adaxial cell material may force the capsules to break open in a premature state in which the lignin ring has not been fully developed yet, leading to visible fruit and seed desiccation (Fig. 3B, C and 4E, H). Interestingly, the premature fruit rupture occurred at random sites along the pericarp, sometimes at the DZ, but also between the median ridges of the valves (asterisks in Fig. 4E, H). The pericarp cells along the rupture were not altered and ectopic lignification did not occur. The patterning of the DZ and the abaxial valve tissues, including sclerenchyma caps and vascular bundles, as well as funiculus and placenta tissue did not seem to be altered drastically in the *EscaSTK-*VIGS treated capsules.

Furthermore, *EscaSTK*-VIGS treated capsules often harbored seeds with irregular shape (Fig. 4E, G, H, J). We observed that endocarp development and seed filling was disturbed in *EscaSTK-*VIGS treated plants. However, the surface cells of those seeds had a turgescent shape as observed in seeds of untreated plants, indicating that seed developmental defects were not simply provoked by early desiccation (Suppl. Figure 5C, D).

In *mEGFP*-VIGS treated seeds as well as with phenotypically less conspicuous *EscaSTK-VIGS* treated seeds, the testa layers derived from the inner and outer integuments were clearly delimited (Fig. 4D, Suppl. Figure 5 D, E). As a typical feature of wild type California poppy seeds, the homogenous outermost epidermis of the seed coat features stomata [24], as do the *EscaSTK*-treated seeds (Fig. 4J, inset). Epidermal cells of the seed coat are differentiated into flat facet cells and elongated, partially multilayered ridge cells (F and R in Fig. 4D, Suppl. Figure 5D), which had not yet collapsed in this developmental stage.

Conversely, we find that in severely affected *EscaSTK*-VIGS treated seeds, discrimination of the testa layers was ambiguous (Fig. 4 G, J). A strikingly thick, uniform parenchyma, composed of mostly five to six layers of relatively large cells formed at the seed surface. In the three more intensely stained cell layers underneath, which presumably emanated from the inner integument, single cell layers could hardly be discriminated, as cells had flattened in radial direction during seed growth.

In summary, we find thick layers of parenchymatous cells lining the adaxial side of the fruit wall of *EscaSTK*-VIGS treated plants that may cause premature fruit rupture. Further, seeds of *EscaSTK*-VIGS treated plants seeds fail to expand and develop additional layers of voluminous cells on the surface. This suggests that *EscaSTK* is involved in restricting cell divisions in the endocarp layer of the capsules, seed filling, and seed coat formation.

### *STK* orthologs are differentially expressed in the dry dehiscent fruits of legumes

**Fig. 5:**
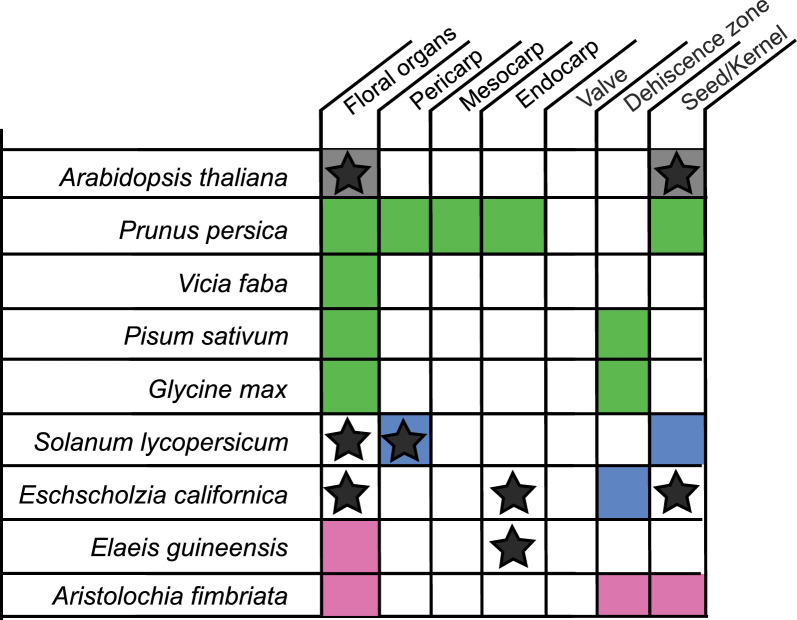
Domains of expression and/or function of *STK* orthologs in angiosperms. Expression of *STK* orthologs in floral organs and fruit tissues are shown in different colors (green for qRT-PCR, pink for *in situ* hybridization, blue for transcriptomic data, grey for all sources) of angiosperm species. Functional studies of *STK,* affecting development of specific tissue are indicated with star symbol. White boxes show lack of expression or no expression/functional data available. Based on: *Arabidopsis thaliana* [33], *Prunus persica* [56], *Solanum lycopersicum* [21, 53], *Elaeis guineensis* [54], *Aristolochia fimbriata* [55]

Legumes are agriculturally important protein crops also producing dry dehiscent fruits, and we were interested if we can find differential expression of *STK* orthologs in the fruits of legumes, providing insights into *STK’s* functional role and its degree of conservation across eudicots. The soy bean genome encodes for two *STK* homologs, termed *GmSTK1* and *GmSTK2*. They show low or no expression in leaves, and substantial expression in buds and open flowers. Both *STK* homologs are expressed in DZs, but only *GmSTK2* is also expressed in early stage of DZ formation. Both genes show no expression in the valves (Fig. 5, Supplemental Fig. 6). The faba bean (*Vicia faba*) and pea (*Pisum sativum*) genomes each encode for a single *STK* ortholog, termed *VfSTK* and *PsSTK*, respectively. *VfSTK* expression is found in leaves, buds and flowers, but hardly in developing fruits (Fig. 5, Supplemental Fig. 6). *PsSTK* shows no expression in leaves and low expression level in buds. However, in open flowers and DZs, it is expressed at a high level. Taken together, our data show that *STK* orthologs are expressed in the fruits of soy and pea, but most likely not in faba bean, suggesting that their possible roles in fruit development may not be fully conserved throughout the legumes. On the other hand, the expression of *STK* ortholog in *Aristolochia fimbriata* at the DZ suggests a potential conserved role in DZ patterning [55]. In contrast, *STK*’s role in ovule identity has been widely demonstrated across various angiosperm species, emphasizing its conserved function in ovule specification (Fig. 5, [33]).

## Discussion

Our study focused on functional analyses of *EscaSTK* regarding DZ patterning in California poppy. Prior, we conducted phylogenetic analyses of known DZ regulators from *Arabidopsis*, generated transcriptomic data from DZ and valve tissues at two distinct developmental stages, and examined the functions of *EscaSTK* through downregulation experiments, analyzing its effects on fruit development. The narrow petal shape observed in the mock control is likely due to the VIGS vectors used in our study. VIGS relies on a viral vector system that may induce mild viral symptoms caused by the host's antiviral stress response [11, 61]. These symptoms can result in developmental changes, including alterations in petal shape or reduced fruit length. Additionally, the limitations of VIGS must be considered, as residual transcript activity may influence the phenotype, making it less reliable than phenotypes coming from knock-out experiments. Nonetheless, VIGS is a valuable genetic tool for non-model plants, providing a foundation towards understanding DZ patterning in California poppy ene regulatory network for the dehiscence zone patterning.

Dry dehiscent fruits require intricate patterning mechanisms allowing for a coordinated fruit rupture in combination with mostly explosive seed dispersal. As dry dehiscent fruits originated independently in several angiosperm lineages, for example within the Rubiaceae (Gentianales, asterids), where transitions from dry to fleshy fruits are frequent [52]. In contrast, transitions vice versa, from fleshy to dry fruits, have occurred within the Solanaceae, but are less frequent.[40]. We were interested in learning if and how the underlying developmental program differs between *Arabidopsis* as a representative of the Brassicaceae and California poppy as a representative of the Papaveraceae, two lineages that split more than 150 million years ago [64]

Our comprehensive phylogenetic analysis of the GRN involved in the patterning of the *Arabidopsis* DZ revealed that many regulators are absent in species outside the Brassicaceae, a finding also supported by [41]. However, the genomes used for data mining in this analysis were of markedly high quality, such that the absence of a gene is not due to lack of sequence information but reflects either gene loss or lineage specific WGD not shared by all taxa in the dataset. It is conceivable that more distant homologs of DZ regulators fulfill similar functions. However, it is likely that sequence divergence causes functional divergence as well [31]. Thus, based on our analysis, we can conclude that the GRN involved in regulating the patterning and development of dry dehiscent fruits in *Arabidopsis* is not conserved throughout angiosperms. More likely, genes regulating patterning of dry dehiscent fruits in the diverse angiosperm lineages were recruited independently.

### Conservation of STK orthologs in seed coat development

Seeds are a major contributor to the colonization of terrestrial habitats by vascular plants. They are vectors for nutrients and water supporting initial seedling growth and provide protection against physical force. The seed coat acts as seed’s interface with the environments and its chemical and physical properties determine the degree of protection and control dormancy and germination [7, 35]. During California poppy fruit development, seeds develop from anatropous, bitegmic ovules, and as such, they do not deviate in their development and morphology from the majority of seeds found in dicots. The seed coat is a maternal, diploid tissue derived from the two integuments surrounding the ovule. The Arabidopsis *STK* gene has an important role in ovule and subsequent seed development, as *stk shp1 shp2* mutants fail to develop both integuments and in their place a structure reminiscent of a carpeloid organ is formed [46]. However, during *Arabidopsis* seed development, the number of layers in the seed coat and their morphological differentiation is similar in wild type and single *stk* seeds, but the cells are smaller [33]. Only double mutant combinations of *STK* and *ARABIDOPSIS BSISTER* (*ABS*) show abnormalities in their cellular differentiation in that they lack the endothelium [34]. This is unlike in the *EscaSTK*-VIGS treated California poppy seeds, whose seed coat shows substantial deviations from control-treated plants in their state of differentiation, number and size of cells. While some layering of the seed coat tissue can be observed (Fig. 4G and J), the outer tissue layers of the *EscaSTK*-VIGS treated plants appear parenchymatous suggesting that seed coat cell identity may not have been fully established in these plants. Apparently, the role of *EscaSTK* as regulator of tissue differentiation in the seed coat is different from that of its *Arabidopsis* ortholog, and independent of the California poppy *ABS* gene.

The identity of this thick layer of parenchymatous tissue surrounding the seed in the *EscaSTK*-VIGS treated plants remains unclear, but a second hypothesis regarding its origin relates to the Arabidopsis *shp1 shp2 stk* ovules that develop carpel-like structures around their integument-free nucellus. In analogy, the tissue surrounding the *EscaSTK*-VIGS treated seeds may also be undifferentiated carpel wall, such that without *EscaSTK*, the ovule integuments undergo homeotic conversions and a carpel is formed instead. The absence of *SHP1* and *SHP2* in genomes outside the core eudicots (Fig. 1B) supports this hypothesis, and possibly, integument identity in California poppy relies on *EscaSTK* alone. However, further analyses are required to support this hypothesis, requiring earlier stages ovules from *EscaSTK* knock-down plants.

Further, *Arabidopsis* *stk* seeds are smaller, due to a smaller cell size in the seed coat, and it was shown previously that *STK* activates several target genes, for example the cell cycle regulator *E2Fa*, influencing cell cycle progression in the developing seed coat by directly binding to its promoter leading to histone modifications [42]. The *EscaSTK*-VIGS treated seeds are also smaller and malformed, but we cannot rule out that their size and shape difference is due to the pressure the massive endocarp is exerting on them. However, Fig. 4 G and J shows that the cell size of *EscaSTK*-VIGS treated seeds outer cell layers seems increased, suggesting that the involvement of *STK* orthologs in cell cycle control seems conserved between the two species, but not the specific molecular mechanism of cell size regulation, resulting in opposing phenotypes. Moreover, *EscaSTK* not only regulates cell size, but also cell divisions in the outer layers of the seed coat, as the parenchymatous tissue is five to six cell layers thick, providing an additional link of *EscaSTK* to cell cycle regulation.

California poppy has two *AGAMOUS* genes, *EscaAG1* and *EscaAG2* which share high sequence similarity with *EscaSTK*. Previous functional analysis revealed that *EscaAG1/2*-VIGS lead to a floral homeotic conversion of stamens into petals, similar to *EscaSTK*-VIGS (Fig. 3A), in which the outer stamen whorl is also converted into petals [59]. Interestingly, *EscaAG1/2* is expressed in the seed coat [60]. Therefore, it is likely that *EscaAG1/2* and *EscaSTK* may have overlapping functions conferring stamen identity and seed coat development. Further qRT-PCR analysis showed no significant down- or up-regulation of *EscaAG1* and *EscaAG2* in *EscaSTK*-VIGS buds (data not shown), suggesting *EscaAG1/2* and *EscaSTK* act redundantly to confer stamen identity. However, it is likely that *EscaAG1/2* may function with *EscaSTK* together for proper seed coat development.

### Conservation of the gene regulatory network for endocarp development

The endocarp protects the seed and is involved in communication with the maturing seed [8]. In *Arabidopsis*, the endocarp is composed of two layers (en*a* and en*b*) which have contrasting functions: en*b*, together with the replum and valve margins accumulates lignin and solidifies, creating tension upon dehydration. En*a* secretes cell wall degrading enzymes creating the separation layer from which the valves separate. While *IND* is directing the lignification of the en*b*, *ALC* activates the cell wall degrading enzymes in the separation layer en*a*. Both genes are prevented to act outside their expression domain by *FUL*, preventing their expression in the valve, and *RPL*, preventing their expression in the replum, as *FUL* and *RPL* target the *SHP1/2* genes which activate *IND* and *ALC* [13, 29, 36]. Within the Brassicaceae, the genetic pathway seems rather conserved, as mutants of the *Arabidopsis* dehiscence pathway orthologs in *Lepidium campestre* have similar phenotypes than those from Arabidopsis [27] and changes to DZ architecture may be due to expression changes rather than absence of regulators.

In core eudicots, the gene *ALC*, a direct target of *SHP* orthologs, is present in multiple lineages, including Brassicaceae and Solanaceae, while *IND*, also a target of *SHP* orthologs, arose through a whole genome duplication and is Brassicales-specific [38]. Functional studies in tomato (*Solanum lycopersicum*) show that a double knock down of *ALC/SPT* leads to ectopic lignification during fruit maturation, suggesting that the lignin-repressing function of *ALC* may be conserved in core eudicots [37]. However, almost all angiosperm genomes included in our analysis encode for *HEC3/IND* genes and *ALC/SPT* genes, which are homologs of the Brassicales *HEC3* and *IND* and the *ALC* and *SPT* orthologs, respectively. Unfortunately, outside core eudicots, most of their functions remain unknown. While *ALC's* function has been studied in Solanaceae, the role of DZ regulators outside *Arabidopsis* is still unclear, except for *PvIND* in common bean, a candidate locus for pod shattering with an unclear mechanism and phenotype (Gioia et al. 2013).

Only few examples exist so far reporting *STK* homolog expression in the endocarp: in *Prunus persica* (peach), expression of the homologs of *SHP* and *STK* in the endocarp suggest that they may be involved in endocarp formation and regulation of its lignification. Conversely, *FUL* homolog expression is found outside the endocarp, possibly to restrict endocarp and limit lignification to its margins [9]. In *Elaeis guineensis* (oil palm), the *STK* ortholog *SHELL* regulates endocarp thickness, and the naturally occurring mutant shows a reduced endocarp size in combination with much higher yield than in the homozygous wild type oil palms. Interestingly, the tomato *STK* ortholog *SlAGL11* is expressed during early fruit development. Overexpression of *SlAGL11* resulted in fleshy sepals, reduced fruit size and locular space [21]. Taken together, *STK* function is not limited to seed development, but is involved in fruit and pericarp development in angiosperms (Fig. 5).

Generally, the pericarp of California poppy fruits is lignified in a pattern different to *Arabidopsis*, and it is most likely the lignified valve ridges intersperse by dehydrating parenchymatous mesocarp cells that create the tension required for detachment of the valves from the replum-like region. Interestingly, the separation layer between the lateral valve ridges and the sclerenchyma of the replum-like region is composed of a narrow layer of cells that are even slightly lignified. Possibly, cell wall degrading enzymes are not required to actively enforce dehiscence, rather, premature rupture is prevented by the formation of a lignified ring all around the fruit very late during fruit maturation (Fig. 2A, Suppl. Figure 2, Suppl. Material 1).

### Unrestricted endocarp growth changes fruit morphology impacting dehiscence

In many dry dehiscent plant species, the differentiation of endocarp layer plays a major role in dehiscence. Shortly before dehiscence occurs in California poppy, the endocarp cell layer dehydrates and shrinks. This shrinkage creates tension in the pericarp, ultimately leading to valve detachment at the DZ. The proliferation of endocarp cells, as observed in *EscaSTK*-VIGS fruits, alters the fruit's anatomy and disrupts the exertion of tension, thereby impeding dehiscence. Our results emphasize that dehiscence is not solely dependent on lignified tissues but instead requires the properly coordinated development of the entire pericarp. Possibly, the specialized role of the endocarp in being a major contributor to dehiscence has originated in the lineage leading to core eudicots, and in species outside the core eudicots, other parts of the pericarp are equally or more important for dehiscence. Dry dehiscent fruits have originated several times independently in several large taxonomical groups, including the Solanaceae, Apiaceae, and Campanulaceae, with frequent transitions back to fleshy or dry, dehiscent fruits [40, 48]. In particular, *SlAGL11* has shown to be involved in development of fleshy tissue [21]. It is thus possible that in several of the fleshy to dehiscent fruit shifts, different parts of the pericarp provided specialized anatomies optimized for dehiscence. Interestingly, even though fruit formation is an important ecological and agronomical trait, research on the molecular evolution of fruit types is rare. More specifically, we lack functional studies documenting the transitions from dry dehiscent to fleshy fruits and vice versa. In most fleshy species, the fleshy part of the fruit is made up often by the parenchymatous mesocarp, but in several crop species, endocarp becomes fleshy, as seen in watermelon or mango [8]. Our study and the work by Pabon-Mora et al., [39] on *EcFUL1* and *EcFUL2* are the first cases where reduction of gene function resulted in additional, and more fleshy cells in the endocarp layer. One could hypothesize this phenotype as a first step towards a transition from dry dehiscent to fleshy fruits, involving two MADS-box transcription factors generally involved in flower and seed formation that may have gained a novel function in endocarp growth regulation. If then either *EcFUL1*/*EcFUL2* or *EscaSTK* is lost, capsules become fleshier.

## Conclusions

Our analysis broadly started out to carry out a presence/absence analysis of the major regulators of dehiscence zone formation in *Arabidopsis*. Finding that many members of the DZ GRN are lacking from species outside the Brassicaceae, we intended to identify dehiscence zone regulators in California poppy, which, like *Arabidopsis*, produces dry dehiscent fruits. The California poppy ortholog of *STK*, *EscaSTK*, which is not involved in DZ formation in *Arabidopsis*, was differentially expressed between DZ and valve. Knock down of *EscaSTK* causes premature rupture of the capsule due to excessive proliferation and elongation of the endocarp cells. Further, *EscaSTK* knock down leads to larger, more, and less differentiated cells in the seed coat, but changes in the lignification pattern of the DZ was not observed in the stages we analyzed. These functional analyses show the remarkable contributions to *EscaSTK* to reproductive development in California poppy: restriction of endocarp cell division and elongation as well as seed coat differentiation and growth limitation. Previous work in *Arabidopsis* demonstrated *STK’s* direct regulation of several pathways associated with seed development, cell cycle and seed specific metabolism. Our work shows that *STK* orthologs are being recruited to several GRNs directing sexual plant reproduction in evolution, such as regulating lignification in the funiculus of ovules, endothelium differentiation and cell size control in the developing seed coat, and activating metabolic pathways in the late stages of seed coat development in *Arabidopsis*. In California poppy, *EscaSTK* contributes to stamen identity, cell cycle control during endocarp and seed coat development. The lineages of *Arabidopsis *and California poppy split around 150 million years ago, sufficient time for a MADS-box transcriptional regulator to diversify its functions, most likely by accumulation of its DNA-binding sites in the regulatory regions of novel target genes and shifts in protein interaction partners. *STK* orthologs may turn up as candidate genes in screens for diverse reproductive traits of traditional or novel crops to become species-specific breeding targets, e.g., for endocarp layer size, seed size or fruit dehiscence.

## Methods

### Plant growth and histology

*E. californica* (cv. Aurantiaca Orange King, B&T World Seeds, Aigues-Vive, France) was grown from seeds at 22 °C under long day conditions (16 h of light, light intensity ~ 95 µmol m-2 s-1). Freshly opened flowers were hand pollinated to ensure fruit development.

Fruits were fixed in FAA (50% EtOH, 5% acetic acid, 10% formaldehyde) plus a drop of TWEEN20 on ice (4 °C) and vacuum infiltrated for 1 h. Fresh FAA was added and the samples were passed through a dehydration series with 30 min in 50%, 70%, 85%, 90% and finally 100% EtOH, respectively, with fresh 100% EtOH overnight incubation at 4 °C. Then followed incubation in 100% EtOH for 1 h, 50% EtOH with 50% Rotihistol for 2 h, 100% Rotihistol for 2 h, and again 100% Rotihistol for 2 h. The fruits were then placed on solid Paraplast Plus chips, covered by Rotihistol and incubated for three days at 60 °C, exchanging the molten Paraplast Plus every 8–12 h. Then the samples were poured into petri dishes allowing the Paraplast Plus to solidify. Samples were sectioned on a Leica RM2125RTS microtome to 5–7 µm thickness, dried, incubated for 20 min in 100% Rotihistol and then rehydrated (5 min steps in 100%, 95%, 85%, 70%, 50%, 30% EtOH, respectively) and dried.

For samples around 18 dap, an alternative embedding and staining protocol was used due to the brittleness of the fruits. The fruits were fixed in 1% PFA and 2% GA in 0.1 M phosphate buffer pH 7.2, followed by 30 min vacuum infiltration and 3 h incubation at RT. The solution was replaced for overnight incubation at 4 °C with gentle shaking. Dehydration was performed as described above with the last step being EtOH:LR-White 1:1 (v/v). After infiltration in 100% LR-White, the samples were placed into gelatin capsules surrounded by resin, polymerized at 50 °C for 24 h and sectioned with a microtome (Leica Om U2, Wetzlar, Germany) to 1–1.5 µm thickness. Sections embedded with the Paraplast method were stained with a 2% (w/v) phloroglucinol–HCl solution (images shown in Fig. 2 A, Suppl. Figure 2A-D and K-M) [47]. Due to the bad staining quality of the phloroglucinol–HCl staining method within LR-White sections, we analyzed these sections via one drop of fuchsin–astra blue diluted in water incubating for 5 min (images shown in Suppl. Figure 2E–J). Alternatively, sections were stained with crystal violet staining solution for better tissue perceptibility. The sections were analyzed using a Leica DM5500B microscope. Images were manipulated for contrast and assembled using CorelDraw12.

### RNA extraction, library preparation, sequencing

Material collection for RNAseq was carried out by manual dissection of 2 and 6 dap fruits to collect valve (v) and dehiscence zone (dz) tissues separately. RNA extraction of fruits of wild type plants for RNAseq and fruits of VIGS-treated plants for qRT-PCR was carried out using the NucleoSpin® RNA Plant kit (Macherey–Nagel, Düren, Germany) according to the manufacturer’s instructions. Library preparation for RNAseq was carried out using the Illumina TruSeqmRNA (San Diego, CA, USA) kit according to the manufacturer’s instructions and sequenced on Illumina NovaSeq6000 (San Diego, CA, USA) at the West German Genome Center (WGGC) Cologne, Germany.

### Phylogeny reconstructions

Protein sequences of dehiscence genes were identified using BLAST [1] to mine the JGI Phytozome database [16], in-house databases (*P. somniferum*, *Vicia faba* [18, 23]*,*), sequences were aligned using MAFFT (–localpair –maxiterate 10,000 –reorder, [26]). Phylogenies (Maximum Likelihood 100 bootstraps) were generated using iqtree (-T AUTO -m TEST -b 100 -con) [32] with full length protein sequences.

### Transcriptome expression analysis

We used the published *Eschscholzia californica* draft genome sequence [20] for transcriptome mapping. The read counts were estimated with Salmon [44], version 1.9.0). The expression levels of orthologs of published genes were used for generating the heatmap. The R package ‘Pheatmap’ was used to generate cluster heatmaps of log-normalized TPM matrices.

### VIGS

A 379-bp fragment of *EscaSTK* including the K-box region was PCR amplified from floral bud cDNA using primers that include EcoRI and BamHI restriction sites, digested, cloned into the equally digested pTRV2 plasmid vector, and sequenced, creating the pTRV2-EscaSTK plasmid. The TRV2-*mEGFP* plasmid included an around 500 bp fragment of *mEGFP* and was prepared the same way. The pTRV1, pTRV2-EscaSTK, and pTRV2-mEGFP vectors were transformed into *Agrobacterium tumefaciens* strain GV3101. Plant infiltration was carried out following the protocol of Wege et al. [58] and Tekleyohans et al., [57]. 155 *E. californica* plants were inoculated with pTRV1 and pTRV2-EscaSTK, 45 plants remained untreated, 30 plants were inoculated with pTRV1 and pTRV2-EscaPDS [58], and 30 plants were inoculated with pTRV1 and pTRV2-mEGFP. Flowers of VIGS-treated plants were hand pollinated. Every seven days, pictures of plants and capsules were taken (12MP 1/2.55″ sensor, 26 mm-equivalent f/1.8-aperture lens, PDAF, OIS), length and width of fruits were measured via ImageJ.

### qRT-PCR

For qRT-PCR, the first floral bud of VIGS-treated plants was collected. cDNA was generated as described above. The relative expression level of *EscaSTK* was determined in untreated and VIGS-treated plants via qRT-PCR. *E. californica* *GAPDH* was used as a reference gene and all primers were tested to have an efficiency above 2. qRT-PCR was carried out using a LightCycler 480 II (Roche, Basel, Switzerland). List of used primers can be found in the Supplementary Table (Suppl. Material 9). Three technical replicates were performed of each sample. qRT-PCR was performed in a total volume of 20 µl containing 10 µl Luna Universal qPCR Mix (NEB),1 µl diluted cDNA (1:50), 1 µl of each primer and 3 µl water. PCR conditions were pre-incubated at 95 °C, followed by 45 cycles of denaturation at 95 °C for 10 s, annealing at 60 °C for 10 s and elongation at 72 °C for 10 s. The relative expression differences was analyzed relative to the wild-typ using the ΔΔCt method [45].

Legume flowers at anthesis, leaves, buds, and fruit dehiscence zones and valves at two developmental stages were collected for qRT-PCR. *Pisum sativum* RNA was isolated using the NucleoSpin® RNA Plant kit (Macherey–Nagel, Düren, Germany) for *Pisum sativum*; *Glycine max* and *Vicia faba* RNA was isolated using the NucleoSpin® RNA Plant and Fungi kit (Macherey–Nagel, Düren, Germany). qRT-PCR was used to detect the expression of *STK* orthologs in different tissues. For *G. max* and *P. sativum*, *ACTIN* was used as housekeeping gene and for *V. faba UBIQUITIN* [19]. Expression was related to these. qRT-PCR and phenotypic data were visualized using the R package 'ggplot2'.

## Supplementary Information

Supplementary material 1: Supplemental Figure 1: Phylogenetic reconstructions of orthologs from Arabidopsis dehiscence zone regulators.

Supplementary material 2: Supplemental Figure 2: Dehiscence zone formation in *E. californica* At 2 dap (A, B), only the xylem of all vascular bundles (vb) in the fruit wall is lignified, but the future sclerenchyma of median valve ridges (mvr), small sclerenchyma caps (sc), lateral valve ridges (lvr), and replum sclerenchyma (rs) is already forming. Lignification pattern has not changed at 6 dap (C, D), but future sclerenchyma cells are more conspicuous, particularly in the dz (D). A discrimination between mvr, sc, lvr, and rs is clearly visible (C, D). Lignification of these sclerenchyma tissues starts at 11 dap and reaches its maximum at 20 dap (E-G: 16 dap, H-J: 20 dap, cf. K-M: 30 dap). Cells in the rs differentiate successively in centrifugal direction (F, asterisk in G). In the replum, thick- walled, but unlignified, cells with a small lumen develop on the adaxial side of the vb (arrow in D). At 16 dap (E-G), radially arranged parenchyma in the valves (E, black arrows) and the parenchymatous separation layers (sl) between the lvr and r in the dehiscence zone (dz) (F and G, black arrows) are non-lignified. At 18 dap, few layers of the radial parenchyma adjacent to the lvr start to develop into lignified sclereids, and this process successively proceeds towards the central mvr of the valves within the next days. Unilateral occurrence of sclereids between central mvr and adjacent sc indicates an intermediate developmental state at 20 dap (I, black arrow), while from 26 dap on, complete valve lignin conjunctions occur between lvr, mvr and the intervening sc (shown in K for 30 dap, black arrow. Sclereids also develop adjacent to the lvr towards the rs in the dz, mainly on the abaxial side (shown in I and J for 20 dap, black arrows). However, the initially parenchymatous sl (I and J for 20 dap, white arrows) becomes lignified only at 30 DAP (cf. L, M, white arrows). Overview of the cross-sectioned mature poppy fruit 32 days after pollination (dap), composed of two valves (v1 and v2) separated by two repla-like tissues (r) in (Q). Each valve includes two sclerified lateral valve ridges (lvr), three sclerified median valve ridges (mvr), and intervening small sclerenchyma caps (sc), covering the adaxial side of the vascular bundles (vb). These sclerenchymatous valve tissues are interconnected via layers of sclereids (scl) traversing the radially arranged parenchyma cells which separate lvr, mvr and sc. Each dehiscence zone (dz) includes two lvr and the central r , which contains a vascular bundle with a lignified replum sclerenchyma cap (rs) and is bordered by narrow, radially orientated lignified separation layers (sl). Ridges, sc, and rs together with sclereids and sl form a closed ring of lignified sclerenchymatous elements, which likely provides mechanic stability to the fruit. Seeds (s) are located within the capsules and are attached to the fruit wall via the placenta tissue at the valve margins. Samples shown in A-D, K-P and Q. were paraffin-embedded and sections were stained with phloroglucinol–HCl, while samples for E-J were embedded in LR-White, which delivered higher quality sections of the dehiscence zone in the intermediate developmental stages. The latter sections were stained with fuchsin–astra blue or crystal violet. e: endocarp, F: facet cells, mvr: median valve ridge, lvr: lateral valve ridge, R: ridge cells, rs: replum sclerenchyma, sc: small sclerenchyma caps; scl: sclereids, vb: vascular bundle Scale bars represent 100 µm in H, K and N, 200 µm in all other images.

Supplementary material 3: Supplemental Figure 3: Heat map of putative developmental regulators of fruit development in California poppy. Transcriptome analysis of 2 dap (young) and 6 dap (later) stage of fruit development showing dehiscence zones separated from valve regions. Clustering was done using Euclidean distance.

Supplementary material 4: Supplemental Figure 4: Phenotypes of *EcPDS*-VIGS treated California poppy plants. Evaluation of *EcPDS*-VIGS phenotyp after silencing the *PDS* gene. Photobleaching phenotype of a whole plant (A), leaves (B), branch including flower bud and leafs (C) and open flower (D).

Supplementary material 5: Supplemental Figure 5: Development of the inner fruit wall tissues in *E. californica*. (A-C) Wild type capsules; (D, E) capsules from *EscaSTK*-VIGS treated plants in cross-section. (A, 16 dap) Stomata (arrowheads) occur in the endocarp layer composed of large cells with thick tangential cell walls, which remain non-lignified throughout capsule development. The arrows point at a sporadically occurring tangential cell division of the endocarp. (B, 20 dap) 4-6 parenchymatous cell layers separate the vascular bundles from the endocarp layer. At the valve margins, the adaxial parenchyma layer(s) bordering the endocarp are slightly enlarged (asterisks in B). Starting at the median region of the valves, endodermal cells and adjacent parenchyma layers collapse successively (B) and this process proceeds towards the valve margins (C, 22 dap). Two clearly delimited testa layers cover the surface of the developing WT seeds (arrowheads in C). They are probably derived from the outer and inner integument of the ovule. Single integument layers could hardly be discriminated, as testa cells had been considerably flattened in radial direction during seed growth. The seed epidermis is still turgescent at this developmental stage and has an irregular outline, due to its differentiation into flat facet cells and elongated, partially multilayered ridge cells (F and R in C). In a phenotypically less conspicuous and closed *EscaSTK-*VIGS treated capsule of the same developmental stage (D), histology of the fruit wall and the seed coat largely resemble that of WT capsules (cf. B, C). Yet, endocarp cells and underlying cell layers at the valve margins, as well as epidermal cells at the seed surface are slightly enlarged. In a severely affected and prematurely opened *EscaSTK-VIGS *treated capsule (E), histology of the dehiscence zone and the abaxial valve tissues largely resembles that of WT capsules in the same developmental stage (cf. B, C). Lignified sclerenchyma of the lateral and median valve ridges, intervening sclerenchyma caps and sclereids, as well as the still non-lignified separation layer (white arrows in B and E) have an ordinary appearance. Apparently, the centrifugal differentiation of the lignified rs is lagging behind, and more non-sclerified cells layers separate the replum sclerenchyma from the outer abaxial fruit epidermis than in WT capsules (compare E to B, C). Yet, the endocarp and several of the adjacent pericarp layers at the adaxial side of the *EscaSTK*-VIGS treated fruit wall are enlarged and strongly elongated in radial direction, so that they fill the locule almost completely. Elongated cells may undergo additional tangential divisions (arrows in E). Bars represent 200 µm e: endocarp, F: facet cells, mvr: median valve ridge, lvr: lateral valve ridge, R: ridge cells, rs: replum sclerenchyma, s: seed, sc: small sclerenchyma caps; scl: sclereids, vb: vascular bundle.

Supplementary material 6: Supplemental Figure 6: Expression of legume *STK* orthologs qRT-PCR analysis of legume flowers at anthesis, leaves, buds, and fruit dehiscence zones and valves at two developmental stages. Log2 normalized expression relative to housekeeping genes are shown. On the top are two *STK* orthologs in *Glycine max, GmSTK1* and *GmSTK2*. The bottom are two orthologs of *STK* in *Vicia faba* and *Pisum sativum*, *VfSTK* and *PsSTK*, respectively.

Supplementary material 7.

Supplementary material 8.

Supplementary material 9.

## Data Availability

Sequence data are provided within supplemental Table 1.
