# Supplementary figures and images for: Conservation of the dehiscence zone gene regulatory network in dicots and the role of the SEEDSTICK ortholog of California poppy (Eschscholzia californica) in fruit development
Source: EvoDevo. 2024 Dec 27;15:16. doi: 10.1186/s13227-024-00236-0 (PMC11673373; doi:10.1186/s13227-024-00236-0)

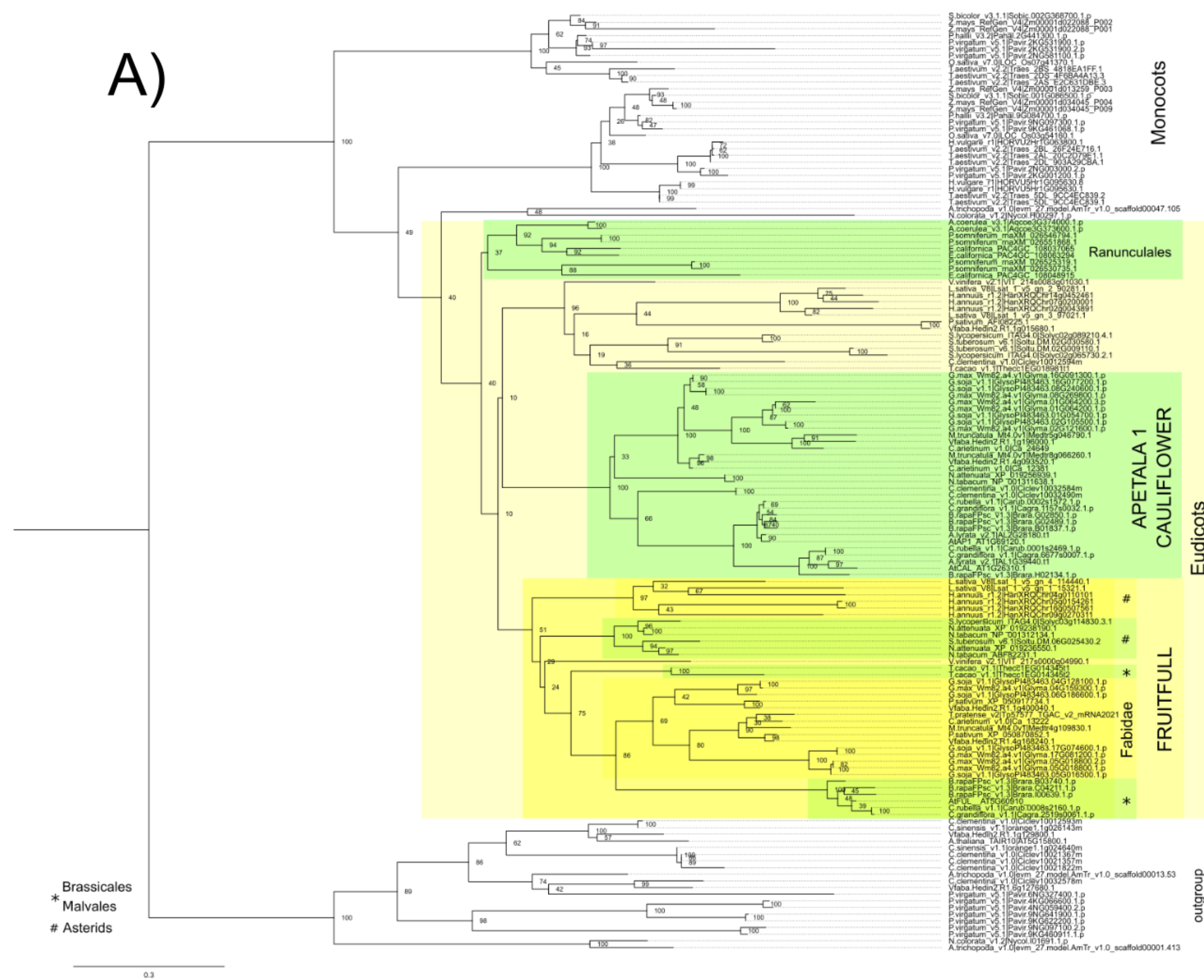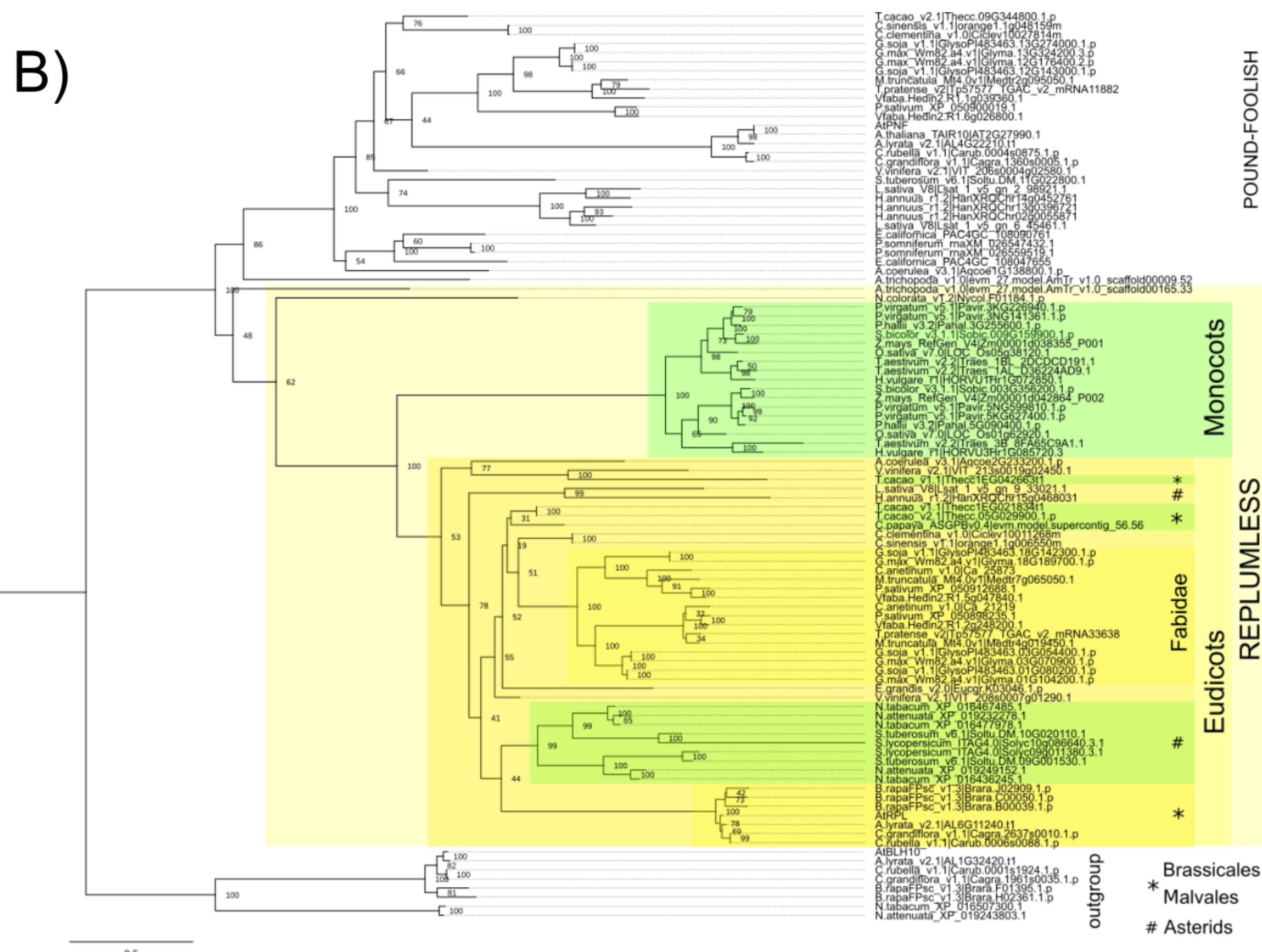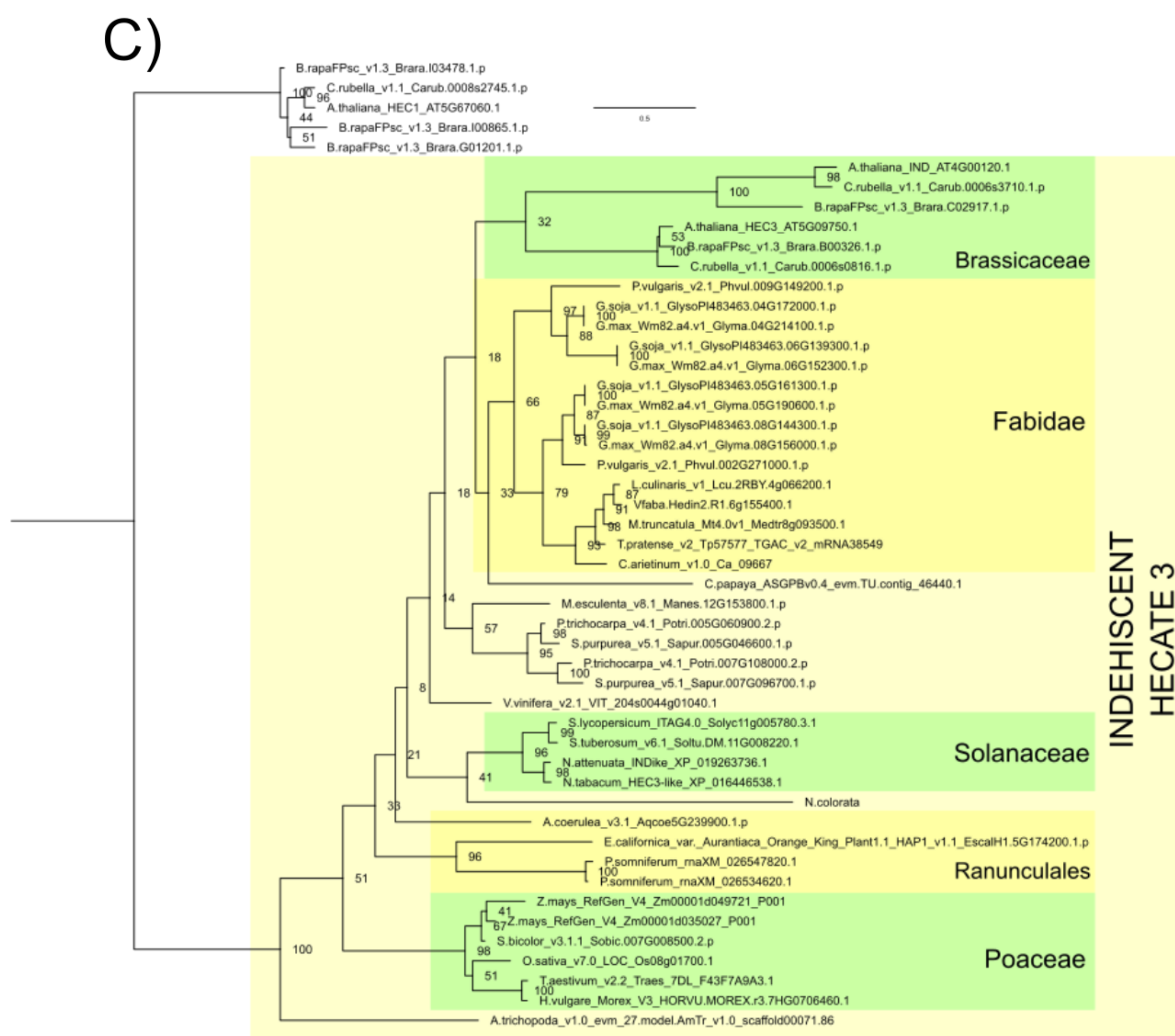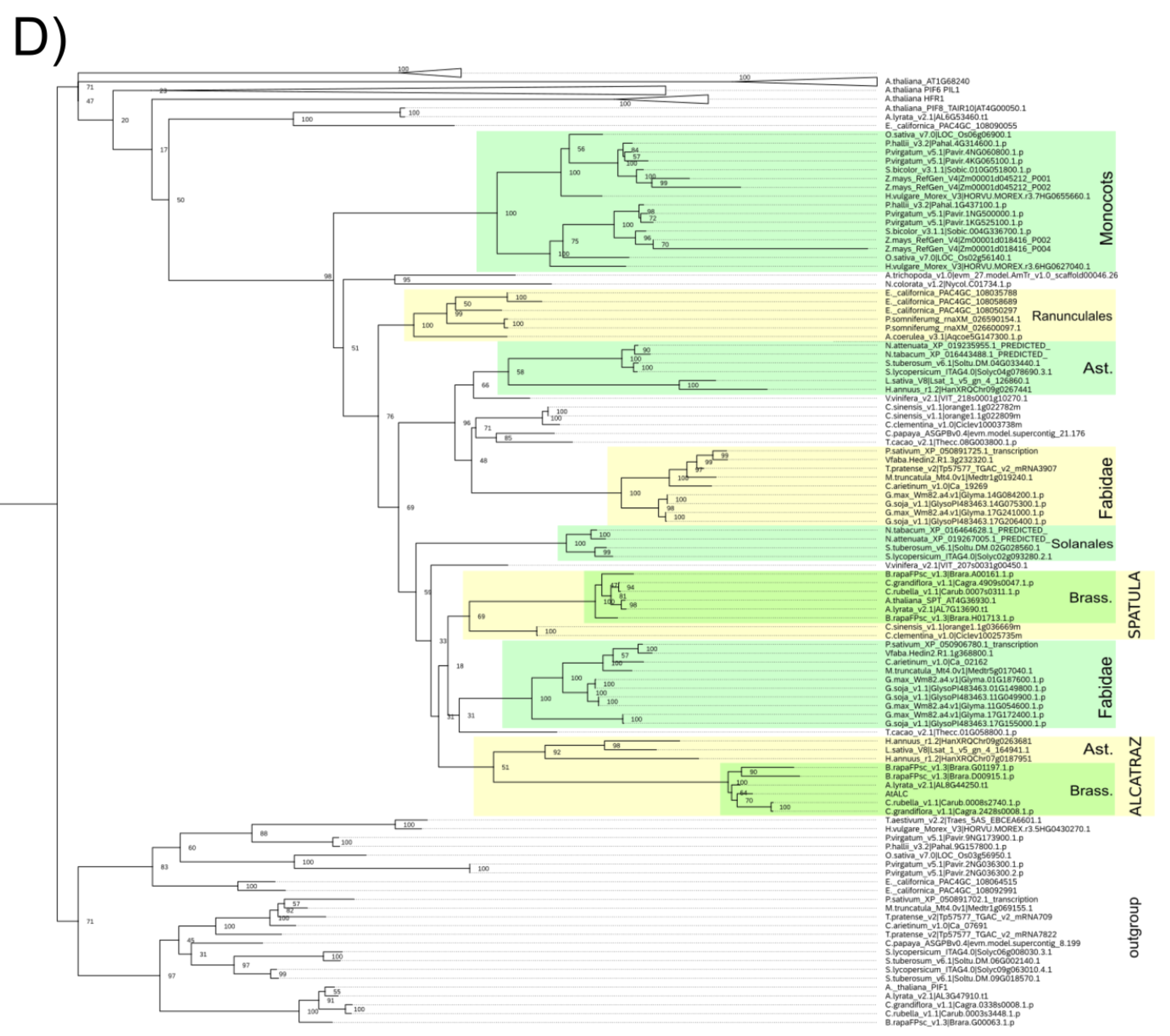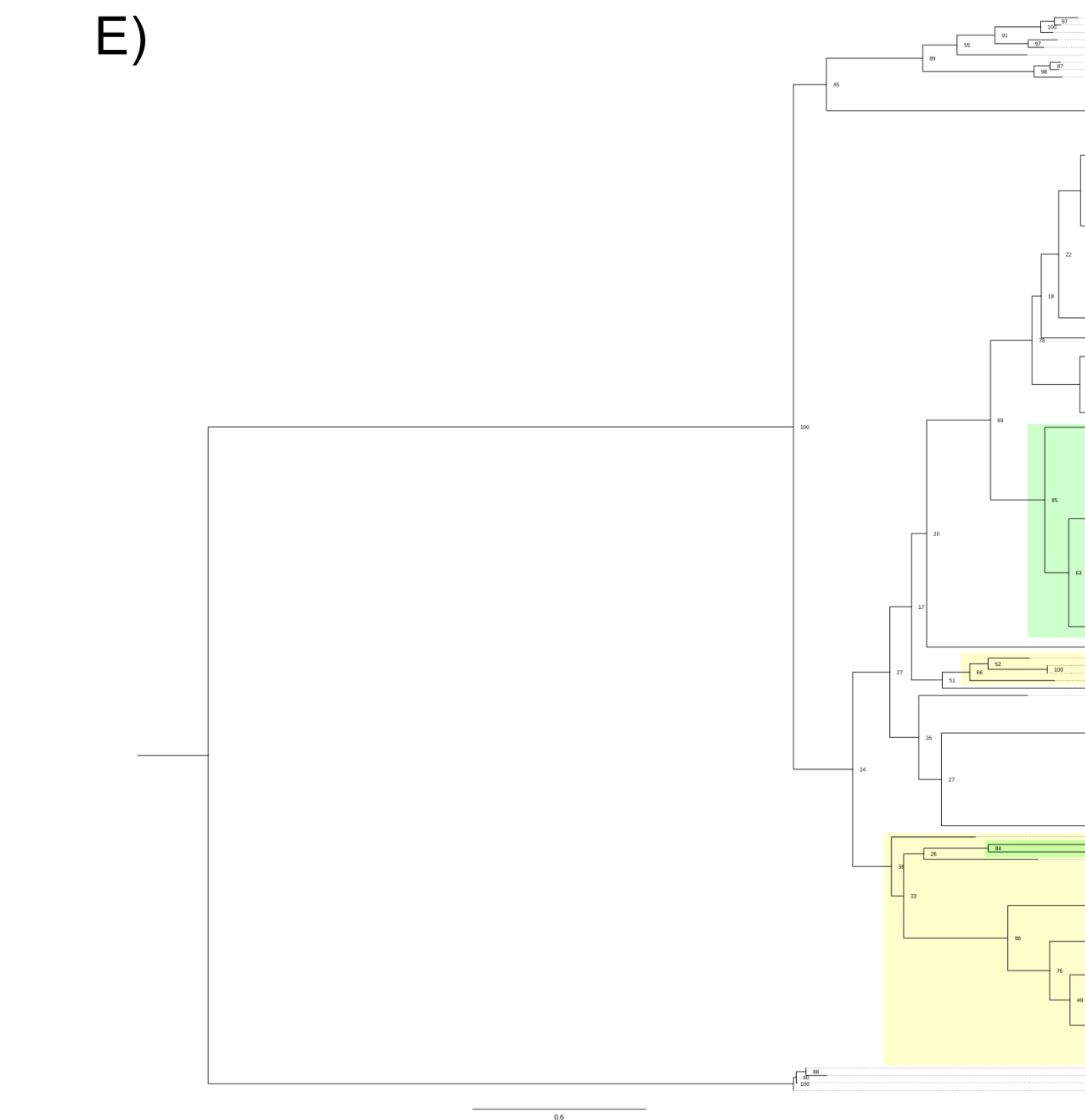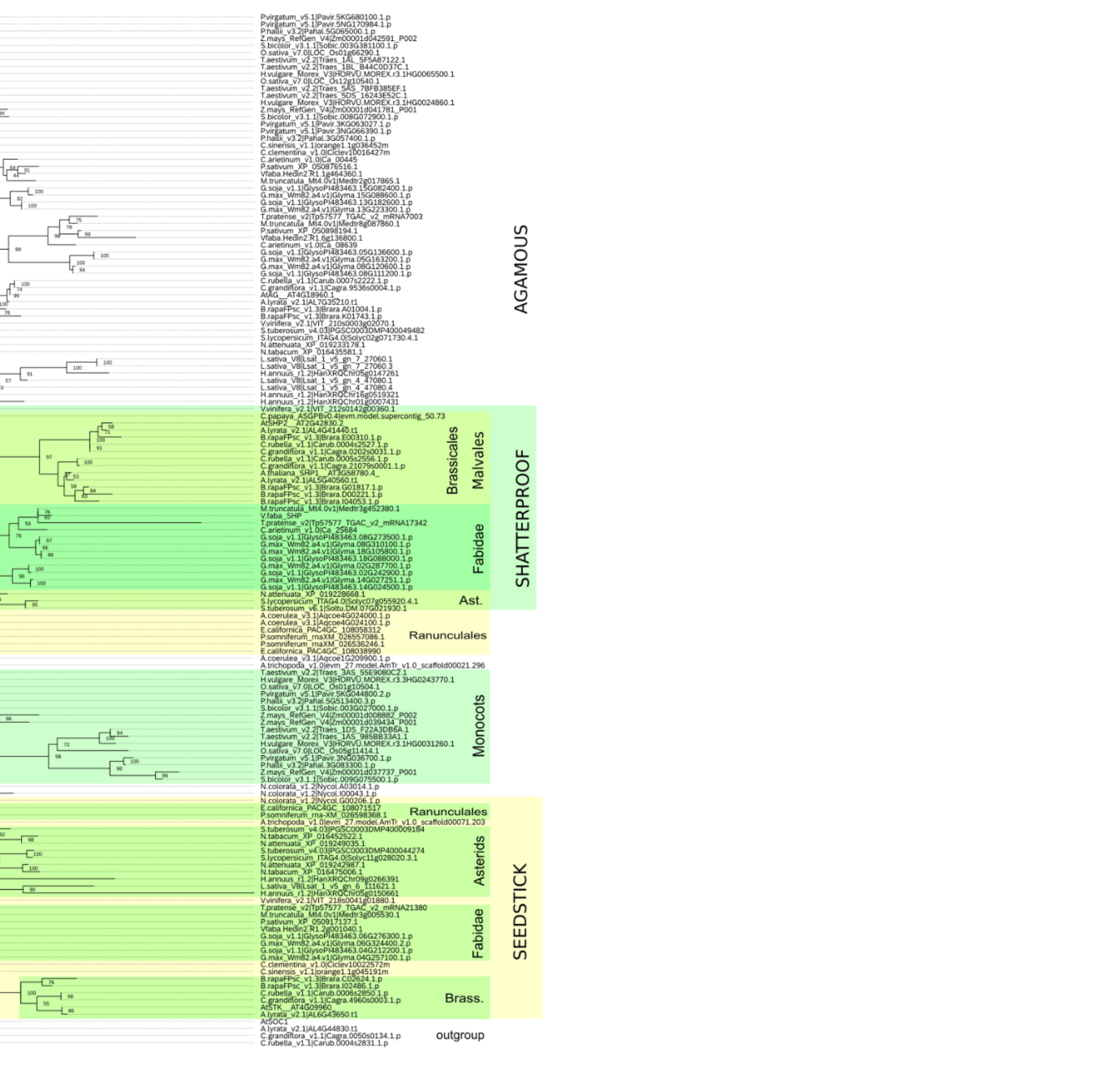

Supplement: Supplementary file 1 — Supplementary material 1: Supplemental Figure 1: Phylogenetic reconstructions of orthologs from Arabidopsis dehiscence zone regulators. [file 13227_2024_236_MOESM1_ESM.pdf]

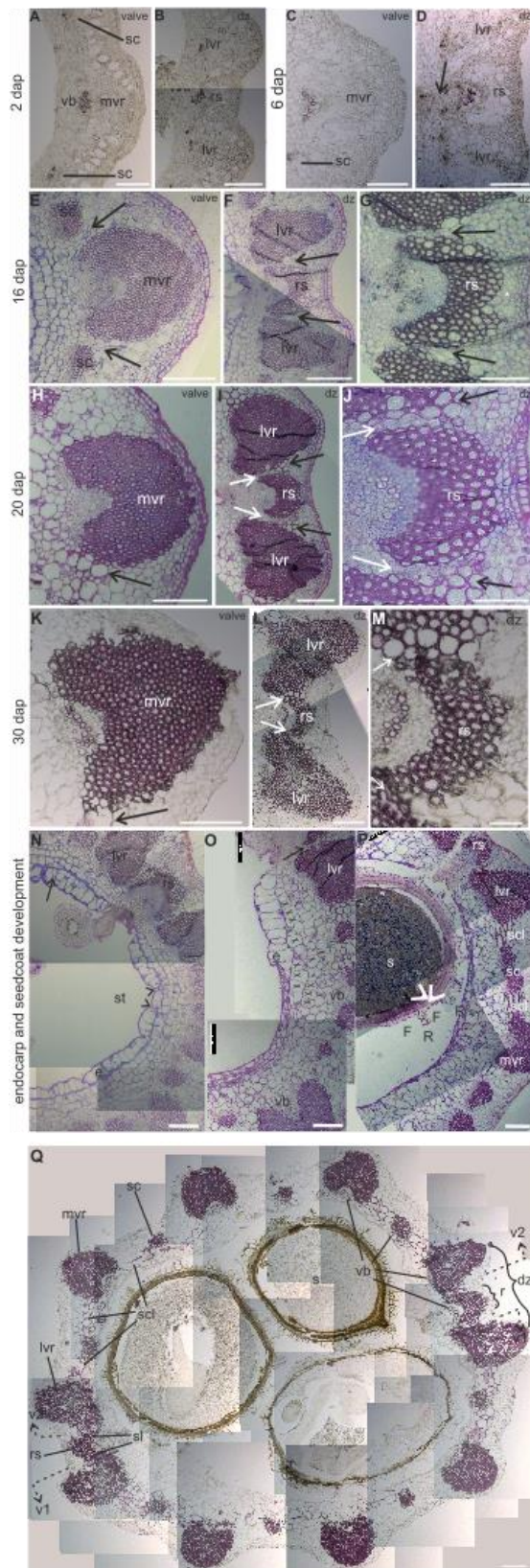

Supplement: Supplementary file 2 — Supplementary material 2: Supplemental Figure 2: Dehiscence zone formation in E. californica At 2 dap (A, B), only the xylem of all vascular bundles (vb) in the fruit wall is lignified, but the future sclerenchyma of median valve ridges (mvr), small sclerenchyma caps (sc), lateral valve ridges (lvr), and replum sclerenchyma (rs) is already forming. Lignification pattern has not changed at 6 dap (C, D), but future sclerenchyma cells are more conspicuous, particularly in the dz (D). A discrimination between mvr, sc, lvr, and rs is clearly visible (C, D). Lignification of these sclerenchyma tissues starts at 11 dap and reaches its maximum at 20 dap (E-G: 16 dap, H-J: 20 dap, cf. K-M: 30 dap). Cells in the rs differentiate successively in centrifugal direction (F, asterisk in G). In the replum, thick- walled, but unlignified, cells with a small lumen develop on the adaxial side of the vb (arrow in D). At 16 dap (E-G), radially arranged parenchyma in the valves (E, black arrows) and the parenchymatous separation layers (sl) between the lvr and r in the dehiscence zone (dz) (F and G, black arrows) are non-lignified. At 18 dap, few layers of the radial parenchyma adjacent to the lvr start to develop into lignified sclereids, and this process successively proceeds towards the central mvr of the valves within the next days. Unilateral occurrence of sclereids between central mvr and adjacent sc indicates an intermediate developmental state at 20 dap (I, black arrow), while from 26 dap on, complete valve lignin conjunctions occur between lvr, mvr and the intervening sc (shown in K for 30 dap, black arrow. Sclereids also develop adjacent to the lvr towards the rs in the dz, mainly on the abaxial side (shown in I and J for 20 dap, black arrows). However, the initially parenchymatous sl (I and J for 20 dap, white arrows) becomes lignified only at 30 DAP (cf. L, M, white arrows). Overview of the cross-sectioned mature poppy fruit 32 days after pollination (dap), composed of two va [file 13227_2024_236_MOESM2_ESM.pdf]

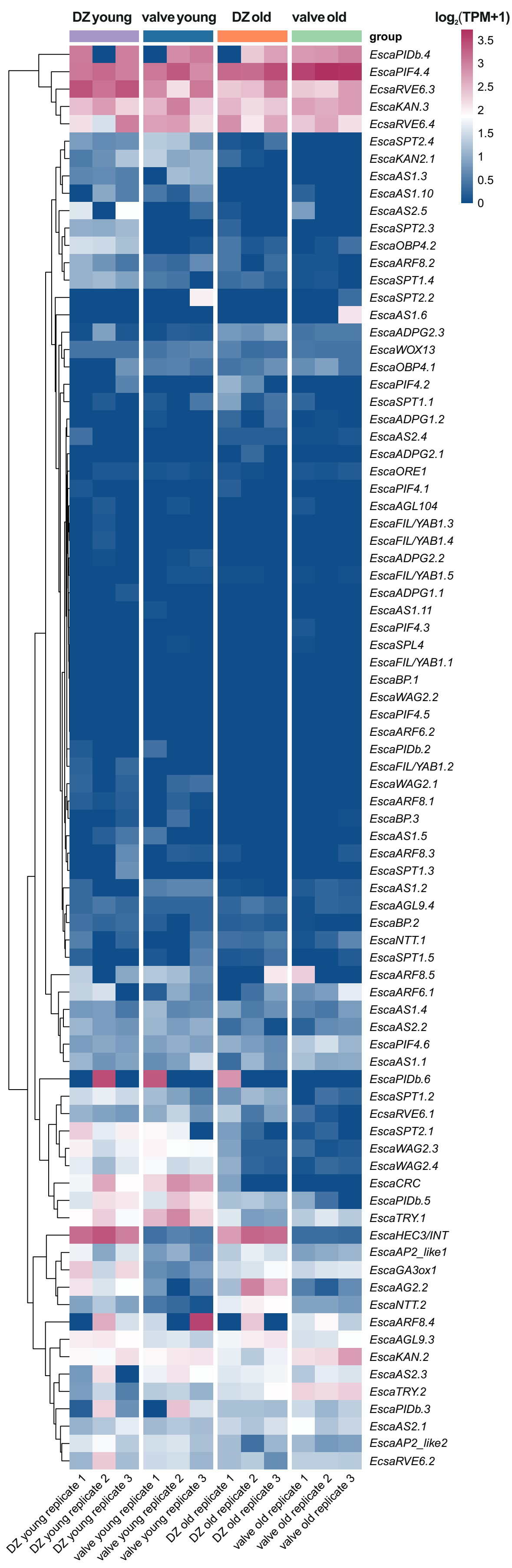

Supplement: Supplementary file 3 — Supplementary material 3: Supplemental Figure 3: Heat map of putative developmental regulators of fruit development in California poppy. Transcriptome analysis of 2 dap (young) and 6 dap (later) stage of fruit development showing dehiscence zones separated from valve regions. Clustering was done using Euclidean distance. [file 13227_2024_236_MOESM3_ESM.pdf]

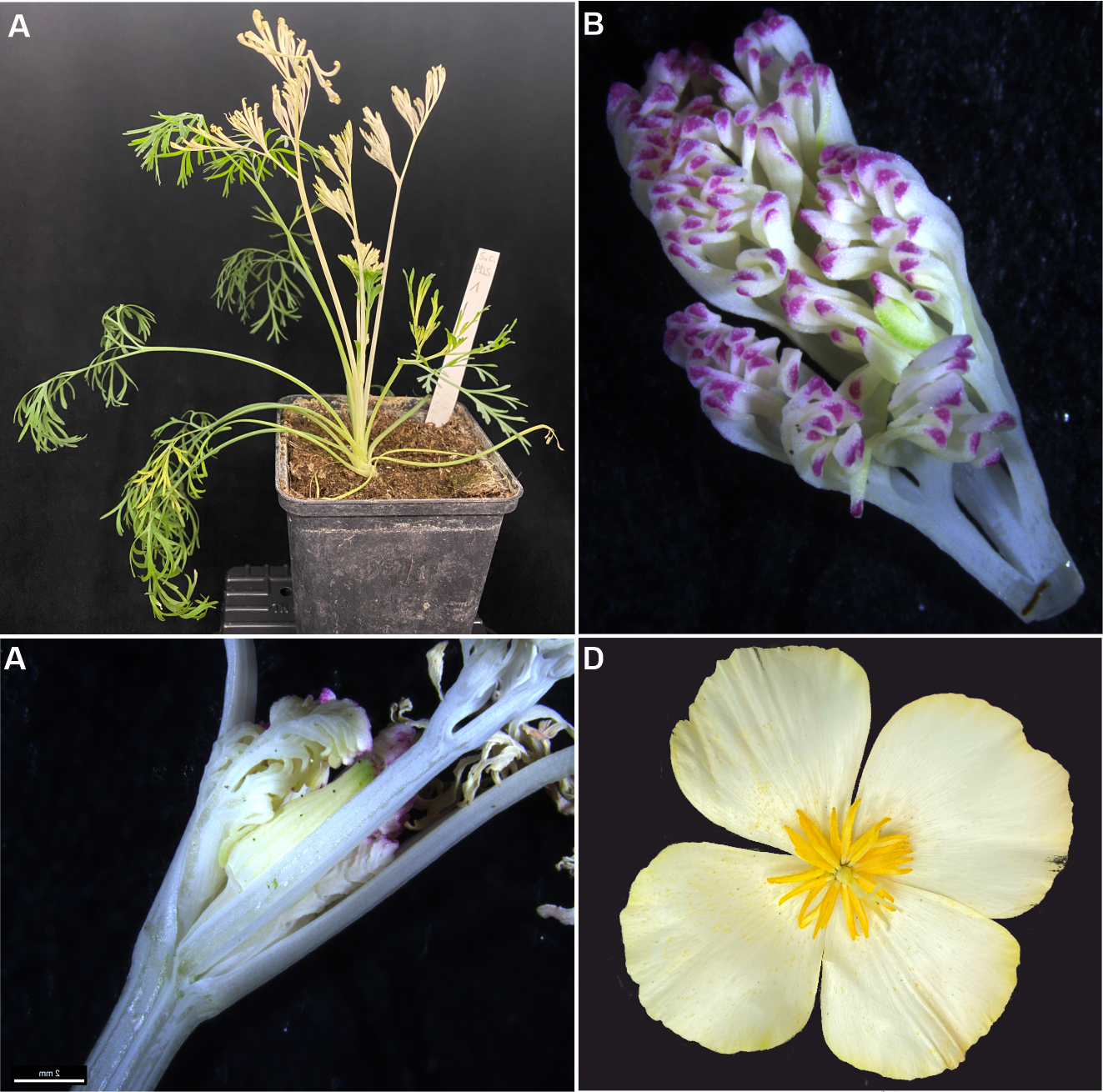

Supplement: Supplementary file 4 — Supplementary material 4: Supplemental Figure 4: Phenotypes of EcPDS-VIGS treated California poppy plants. Evaluation of EcPDS-VIGS phenotyp after silencing the PDS gene. Photobleaching phenotype of a whole plant (A), leaves (B), branch including flower bud and leafs (C) and open flower (D). [file 13227_2024_236_MOESM4_ESM.tif]

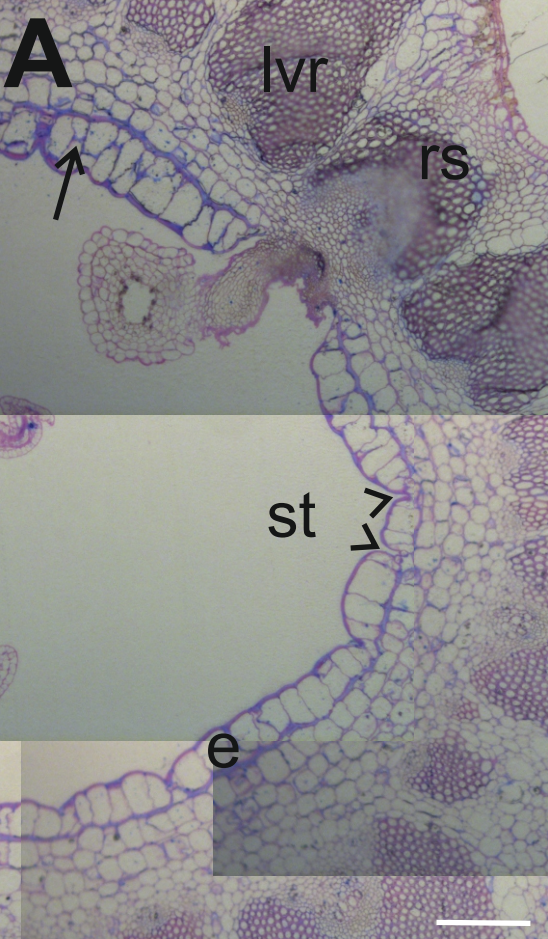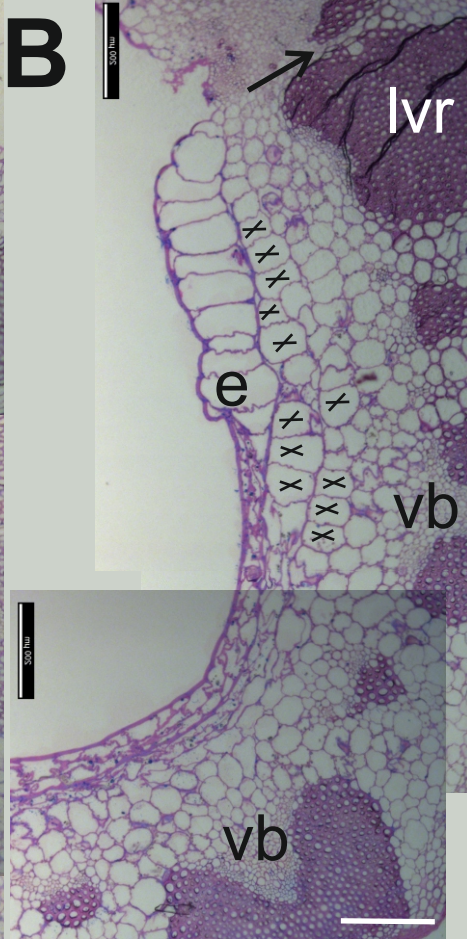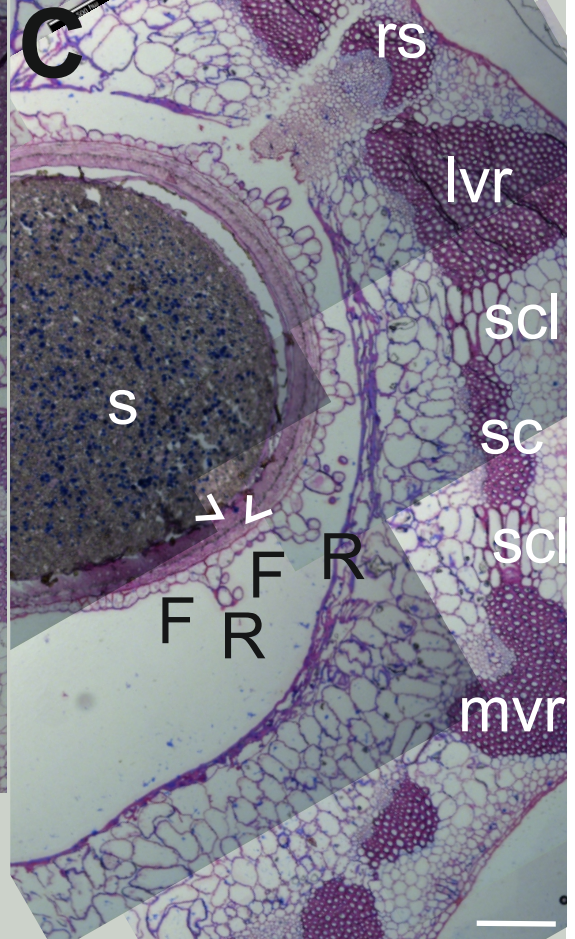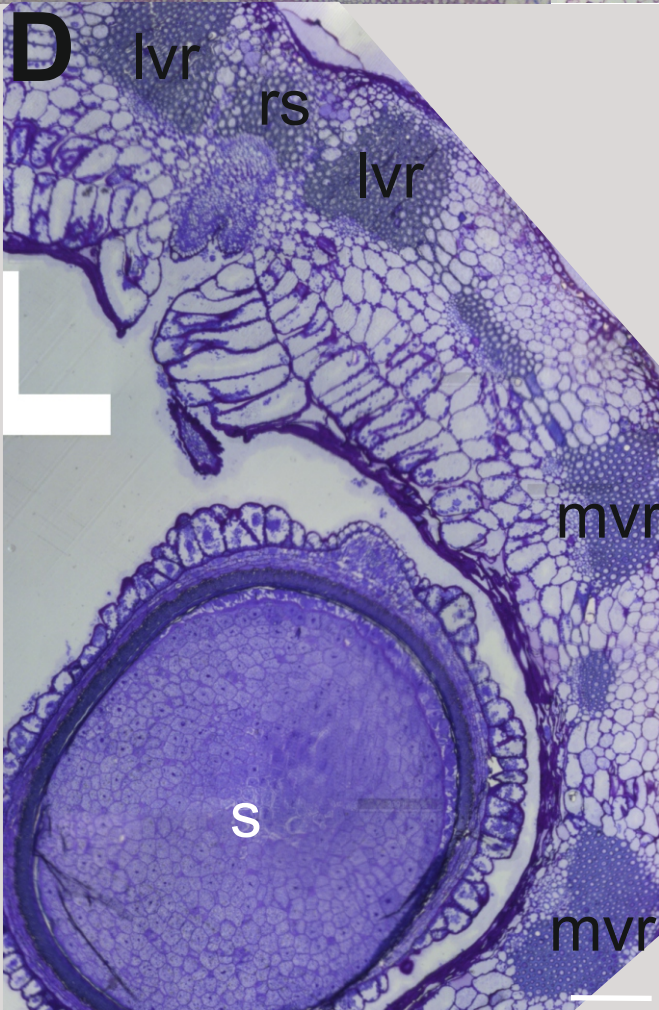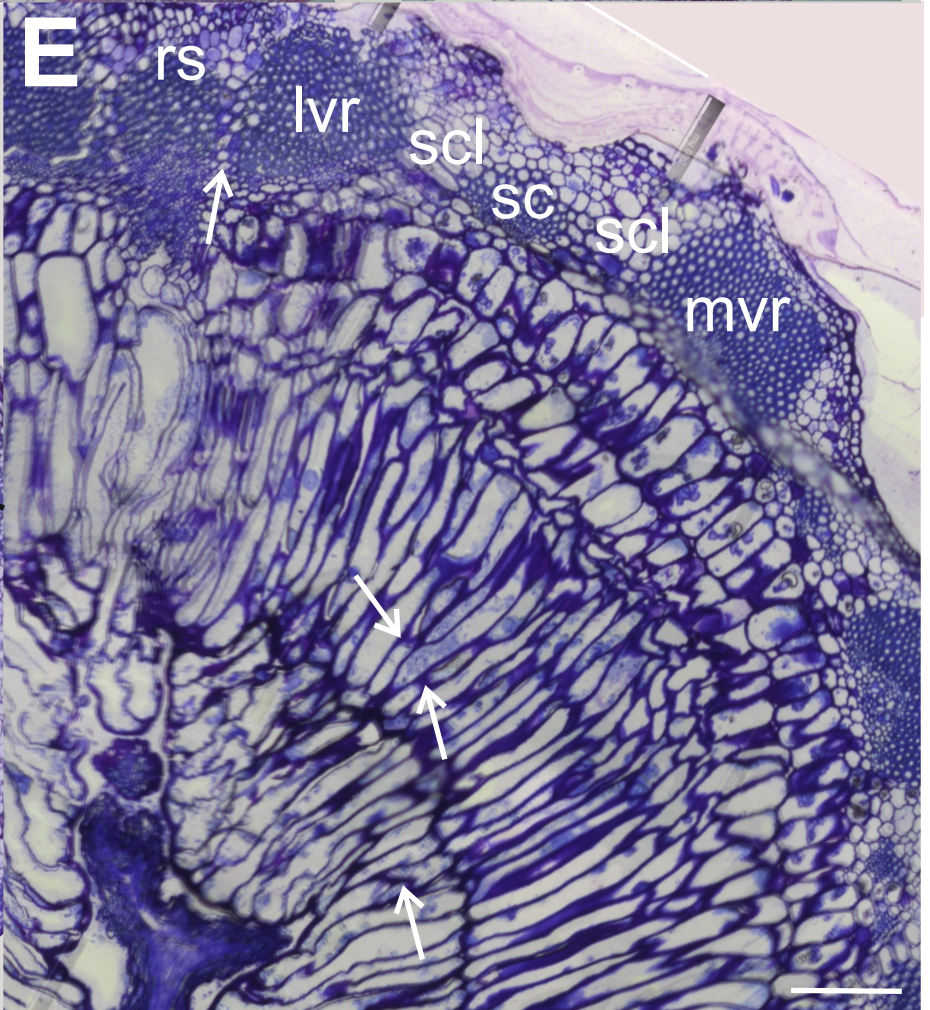

Supplement: Supplementary file 5 — Supplementary material 5: Supplemental Figure 5: Development of the inner fruit wall tissues in E. californica. (A-C) Wild type capsules; (D, E) capsules from EscaSTK-VIGS treated plants in cross-section. (A, 16 dap) Stomata (arrowheads) occur in the endocarp layer composed of large cells with thick tangential cell walls, which remain non-lignified throughout capsule development. The arrows point at a sporadically occurring tangential cell division of the endocarp. (B, 20 dap) 4-6 parenchymatous cell layers separate the vascular bundles from the endocarp layer. At the valve margins, the adaxial parenchyma layer(s) bordering the endocarp are slightly enlarged (asterisks in B). Starting at the median region of the valves, endodermal cells and adjacent parenchyma layers collapse successively (B) and this process proceeds towards the valve margins (C, 22 dap). Two clearly delimited testa layers cover the surface of the developing WT seeds (arrowheads in C). They are probably derived from the outer and inner integument of the ovule. Single integument layers could hardly be discriminated, as testa cells had been considerably flattened in radial direction during seed growth. The seed epidermis is still turgescent at this developmental stage and has an irregular outline, due to its differentiation into flat facet cells and elongated, partially multilayered ridge cells (F and R in C). In a phenotypically less conspicuous and closed EscaSTK-VIGS treated capsule of the same developmental stage (D), histology of the fruit wall and the seed coat largely resemble that of WT capsules (cf. B, C). Yet, endocarp cells and underlying cell layers at the valve margins, as well as epidermal cells at the seed surface are slightly enlarged. In a severely affected and prematurely opened EscaSTK-VIGS treated capsule (E), histology of the dehiscence zone and the abaxial valve tissues largely resembles that of WT capsules in the same developmental stage (cf. B, C). Lignified sclerenchyma of th [file 13227_2024_236_MOESM5_ESM.pdf]

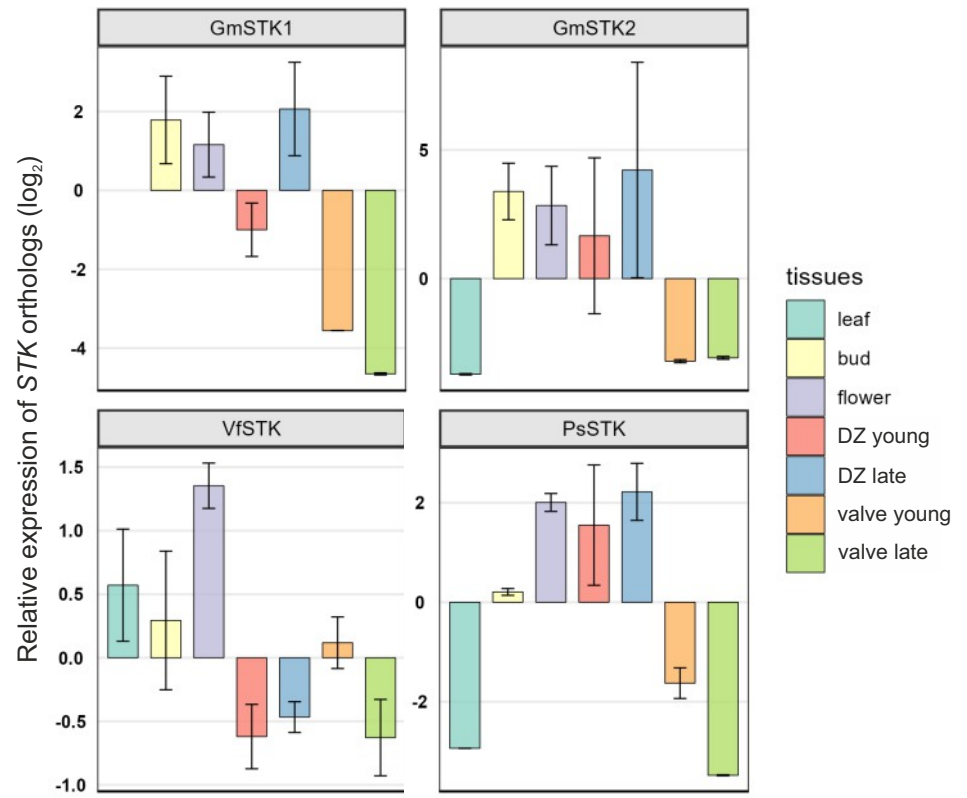

Supplement: Supplementary file 6 — Supplementary material 6: Supplemental Figure 6: Expression of legume STK orthologs qRT-PCR analysis of legume flowers at anthesis, leaves, buds, and fruit dehiscence zones and valves at two developmental stages. Log2 normalized expression relative to housekeeping genes are shown. On the top are two STK orthologs in Glycine max, GmSTK1 and GmSTK2. The bottom are two orthologs of STK in Vicia faba and Pisum sativum, VfSTK and PsSTK, respectively. [file 13227_2024_236_MOESM6_ESM.pdf]
