# Supplementary material for: Conservation of the dehiscence zone gene regulatory network in dicots and the role of the SEEDSTICK ortholog of California poppy (Eschscholzia californica) in fruit development
Source: EvoDevo. 2024 Dec 27;15:16. doi: 10.1186/s13227-024-00236-0 (PMC11673373; doi:10.1186/s13227-024-00236-0)
Supplement: Supplementary file 7 — Supplementary material 7. [file 13227_2024_236_MOESM7_ESM.docx]

**Supplemental Material 1**

**Morphogenesis of the dehiscence zone in *Eschscholzia californica* fruits**

To broaden our knowledge of the dz formation in California poppy, we analyzed the successive lignification events in WT capsules by establishing a time series from 2 to 32 dap with 2-day-intervals of sample collections. Based on our observations, we distinguished six key lignification events starting from xylem lignification in vascular bundles (vb), until a closed lignified tissue ring through the entire fruit was observed in cross sections (see table 1).

| **Event** | **dap** | **Description** |
| --- | --- | --- |
| I | 2 – 10 | Xylem of the vascular bundles is lignified |
| II | 11 – 14 | Start of lignification of cap-like sclerenchyma covering the vascular bundles in replum and valves |
| III | 15 – 20 | Increment of sclerenchyma lignification |
| IV | 18+ | Formation of lignified sclereids from intervening parenchyma between the sclerenchyma caps starting at the replum/valve interface |
| V | 26 | Complete conjunction of lignified tissues in the valves, connecting lateral and median ridges with the intervening small sclerenchyma caps via sclereids |
| VI | 28+ | Lignification of the separation layers at the dz and closure of the ring of lignified tissue |

Table 1 Summary of six lignification events during fruit development in E. californica

During the early time frame (2-10 dap), lignification was only observed in the adaxial xylem tissue of the vascular bundles in the fruit wall, which were circularly arranged and radially separated by parenchyma. Adjacent to each vascular bundle in abaxial direction, a cap-shaped cluster of future sclerenchyma fibers became visible at 2 dap (Suppl. Fig. 2 A and B). These cells later maturated to form the stabilizing lateral and median valve ridges (lvr and mvr, respectively) alternating with smaller sclerenchyma caps (sc) in the valve region, and also the replum sclerenchyma (rs) in the dz (Fig. 2 A). At 6 dap, the sclerenchyma had further differentiated - particularly in the dz - and could be more clearly identified, but no progression of lignification was observed in the valve or dz regions (Suppl. Fig. 2 C and D). In the replum (r), thick-walled cells with a small lumen also start to differentiate on the adaxial side of the vb (Suppl. Fig. 2 D arrow) which, however, remained unlignified throughout fruit development (cf. Fig. 2 A).

As fruit maturation continued, observation of paraplast sections was hindered by rupture of only the dz sclerenchyma, which occurred already at 10 dap and preceded the onset of detectable lignification of all sclerenchyma caps in the fruit. This lignification event was detected by phloroglucinol staining only after 11 dap and proceeded evenly (Suppl. Fig. 2 E-G for 16 dap) until the maximum intensity of staining was reached at 20 dap (Suppl. Fig. 2 H-J for 20 dap and K-M for 30 dap). Particularly in the rs, sclerenchyma cells differentiated successively in centrifugal direction (Suppl. Fig. 2 F, G, and I, J).

Up to and including 16 dap, however, radially arranged parenchyma cells between the bundles and caps in the valves (Suppl. Fig. 2 E, black arrows) stayed unstained, and also the separation layers (sl) between the lvr and the rs in the dz remained non-lignified (Suppl. Fig. 2 F and G, black arrows). Thereafter, few tangential layers of the radial parenchyma cells became enlarged and developed into lignified sclereids (Suppl. Fig. 2 H, black arrow) starting synchronously at all lvr and proceeding towards the centers of both valves. At 26 dap, these sclereids had successively interconnected lvr, mvr and the intervening sc to complete lignified tissue conjunctions in the valves (Fig. 2 A and Suppl. Fig. 2 K). Adjacent to lvr, some sclereids were also formed towards the sl in the dz, mainly on the abaxial side where the rs is located Suppl. Fig. 2 I, J). However, the proper sl remained parenchymatous until 30 dap (Suppl. Fig. 2 L and M, white arrows) and was the last tissue to become lignified, so that finally a closed lignified tissue ring through the entire mature fruit was observed (Fig. 2 A).

Since lignification events in the endocarp layer were reported to play an important role in fruit scattering e.g. in Brassicaceae (Hofhuis et al., 2016), we also focused on the development of the innermost cell layer in *E. californica* WT capsule walls (Suppl. Fig. 2 N-P). The endocarp layer possessed stomata (arrowheads in Suppl. Fig. 2 N) and was composed of large cells with thick, but non-lignified, tangential walls at 16 dap. At the valve margins, also the inner layer(s) of the 4-6 parenchyma layers adaxial of the vascular bundles became slightly enlarged (asterisks in Suppl. Fig. 2 O for 20 dap). During fruit maturation, endodermal cells and adjacent parenchyma layers collapsed successively starting at the median regions of the valves (Suppl. Fig. 2 O, 20 dap) and proceeding towards the valve margins (Suppl. Fig. 2 P, 22 dap). Lignification of the inner tissues of the capsule wall was never observed at any developmental stage (cf. also Fig. 2 A). In contrast to the development of the closed lignified tissue ring in the *E. californica* capsules described above, chronology of the development of the inner fruit-wall tissues was not strictly synchronized or correlated to the dap. It might rather be influenced by mechanical effects caused by the progress of seed growth.

References

Hofhuis et al., 2016 (https://doi.org/10.1016/j.cell.2016.05.002)
